# Supplementary material for: Putting BASIL in a BLT: A Bayesian filtering method for estimating the fitness effects of nascent adaptive mutations
Source: PLoS Comput Biol. 2026 Feb 27;22(2):e1013946. doi: 10.1371/journal.pcbi.1013946 (PMC12974954; doi:10.1371/journal.pcbi.1013946)
Supplement: S1 Text — (PDF) [file pcbi.1013946.s001.pdf]

Supplementary Materials for

Putting BASIL in a BLT: A Bayesian Filtering Method for Estimating the Fitness Effects of Nascent Adaptive Mutations

Huan-Yu Kuo, Sergey Kryazhimskiy*

*E-mail: skryazhi@ucsd.edu

Contents

1 Estimation of measurement noise variance in the calibration experiment 3

2 Existing methods for the analysis of BLT data 4

2.1 Review of the Levy-Blundell method 4
2.2 Review of FitMut2 6
2.3 Key assumptions of the Levy-Blundell method 8
2.3.1 Assumption 1. Low-abundance lineages are neutral 9
2.3.2 Assumption 2. Read frequency dynamics faithfully represent lineage frequency dynamics 9

3 BASIL 13

3.1 Model of measurement 13
3.2 The belief distribution for lineage size and fitness 15
3.2.1 Projecting the belief distribution 15
3.2.2 Updating the belief distribution 18
3.3 Estimating population's mean fitness and the noise parameter 23
3.4 Identification of adapted lineages and estimation of their selection coefficients 27
3.5 Implementation 29

4 Application of BASIL to simulated data 30
4.1 Belief distribution and lineage identification 30
4.2 Performance on simulated data 30

Appendices 32

A Expectation of the noise variance estimator and bias correction 32

B Expected frequency given the number of reads 33

C Expectation of the logarithm of a gamma-distributed random variable 35

1 Estimation of measurement noise variance in the calibration experiment

Denote by X_p the set of barcodes with the same target frequency p. Variation in the number of reads r_{bi} across barcodes $b \in X_p$ and replicates i arises from three sources. First, the same barcode is represented by different numbers of reads in different replicates since replicates receive different total coverage. Specifically, if the barcode's true frequency in the pool is p_b, we expect to obtain $p_b R_i$ reads for this barcode in replicate i with total coverage R_i. Second, the actual number of reads obtained for barcode b will deviate from $p_b R_i$ by some random amount δr_{bi} due to noise during library preparation and sequencing [1]. We refer to δr_{bi} as "measurement noise". Finally, while we aimed to seed the barcoded clones within the same frequency class X_p with exactly the same target frequency p, in reality, the frequency p_b of barcode in the pool actually deviates from p by some amount δp_b, i.e., $p_b = p + \delta p_b$. We refer to δp_b as the "experimental noise". Putting all of this together, we can model the read count r_{bi} for barcode $b \in X_p$ as

$$r_{bi} = p_b R_i + \delta r_{bi}, \tag{S1}$$

where p_b is unknown and we assume that all δr_{bi} are independent random variables with zero mean, and all δr_{bi} with the same b are identically distributed with variance σ^2_{pi} (the noise variance) where index p indicates that noise variance can vary between frequency classes X_p, and index i indicates that it can also vary between replicates i. Our goal is to estimate σ^2_{pi}.

The challenge with estimating σ^2_{pi} is that each barcode has an unknown expected number of reads $p_b R_i$ in each replicate i. If all barcodes had exactly the same frequency p, then we could use r_{bi} from different barcodes in the same replicate to estimate $p_b R_i$ as

$$\bar{r}_{pi} = \frac{1}{|X_p|} \sum_{b \in X_p} r_{bi}. \tag{S2}$$

However, different barcodes $b \in X_p$ in fact have different frequencies p_b, and the estimate (S2), which ignores this variation, would provide a poor estimate for the expected barcode count $p_b R_i$ and risks inflating our estimate of the measurement noise. To overcome this problem, we first estimate p_b as

$$\hat{p}_b = \frac{1}{n_{\text{rep}}} \sum_{j=1}^{n_{\text{rep}}} \frac{r_{bj}}{R_j}.$$

Then, we estimate the expected number of reads $\bar{r}_{bi}$ for barcode b in replicate i as

$$\bar{r}_{bi} = \hat{p}_b \, R_i = \frac{R_i}{n_{\text{rep}}} \sum_{j=1}^{n_{\text{rep}}} \frac{r_{bj}}{R_j}. \tag{S3}$$

We can now use expectation (S3) to estimate the variance in the read count due to measurement noise in frequency class X_p and replicate i as

$$\hat{\sigma}_{pi} = \frac{1}{|X_p|} \sum_{b \in X_p} \left(r_{bi} - \bar{r}_{bi} \right)^2 . \tag{S4}$$

In Appendix A, we derive the expectation for this estimator. We find that this estimator is biased, approximately by a factor $1 - 1/n_{\text{rep}}$ and derive a bias-corrected estimate (see equation (S73)). However, this corrected estimate is not guaranteed to be non-negative, and indeed we found that the variance estimate for one of the replicates in the lowest frequency class becomes negative upon applying this correction. We also found that for 36 out of 41 (88%) values of $\hat{\sigma}_{pi}$ that we could estimate, the correction changes the estimated value by less than 25%. For these reasons, we decided to use the uncorrected estimate given by equation (S4) for the subsequent analysis.

2 Existing methods for the analysis of BLT data

Levy et al developed the first computational method for the analysis of barcode lineage tracking (BLT) data [2], which we refer to as the "Levy-Blundell" method, or the "LB method" for short. More recently, Li et al developed its extension, called FitMut2, and showed that it is superior to the LB method [3]. In this section, we briefly review both the original LB method and FitMut2.

2.1 Review of the Levy-Blundell method

The LB method is based on a stochastic birth-death process that describes a mutant lineage starting from n_0 founding cells that randomly divide and die with certain per capita birth rate b and death rate d. Levy et al derived the full stochastic expression for the probability $P_t(n)$ that the number of cells in the mutant lineage after time t is n (see equation (126) in the Supplementary Information to Ref. [2]). They then approximate this discrete distribution for large n by a continuous probability distribution density

$$p_t(n) = \frac{1}{\kappa_t} \sqrt{\frac{\mu_t}{n}} \exp\left[-\frac{n + \mu_t}{\kappa_t} \right] I_1\left(\frac{2\sqrt{n \mu_t}}{\kappa_t} \right) \tag{S5}$$

$$\approx \sqrt{\frac{\mu_t^{1/2}}{4\pi\, \kappa_t\, n^{3/2}}} \exp\left[-\frac{(\sqrt{n} - \sqrt{\mu_t})^2}{\kappa_t} \right] , \tag{S6}$$

where $I_1(x)$ denotes the modified Bessel function of the first kind. They then use the approximated expression for the analysis of their data (see equation (127) in the

Supplementary Information to Ref. [2])). Equation (S6) depends on two parameters: the mean of lineage size $\mu_t = n_0 e^{\lambda t}$ and the variance to mean ratio $\kappa_t = \frac{1}{2\lambda}(e^{\lambda t} - 1)$, where $\lambda = b - d$ is the mutant growth rate. One important difference between the full solution of the birth-death process and the approximation (S6) is that the full probability distribution has a non-zero weight at $n = 0$ (i.e., extinction is possible) whereas in the approximation (S6) the probability of extinction is zero.

The LB method then uses the same general form of equation (S6) to describe the dynamics of barcode read counts, that is, if a lineage with selection coefficient s is observed to have r_{k-1} read counts at the previous cycle $k-1$, then the probability density $p(r)$ that r counts will be observed at the next cycle is assumed to be

$$p(r; \mu_k, \kappa_k) = \frac{1}{\kappa_t}\sqrt{\frac{\mu_t}{r}}\exp\left[-\frac{r+\mu_t}{\kappa_t}\right] I_1\left(\frac{2\sqrt{r\mu_t}}{\kappa_t}\right) \tag{S7}$$

$$\approx \sqrt{\frac{\mu_k^{1/2}}{4\pi\,\kappa_k\, r^{3/2}}}\exp\left[-\frac{(\sqrt{r}-\sqrt{\mu_k})^2}{\kappa_k}\right], \tag{S8}$$

where

$$\mu_k(s, \bar{s}_k) = \frac{R_k}{R_{k-1}}\, r_{k-1}\, e^{(s-\bar{s}_k)(t_k - t_{k-1})}, \tag{S9}$$

is the expected read count. Here R_{k-1} and R_k are the total read depths at sampling times t_{k-1} and t_k respectively and $\bar{s}_k$ is the mean fitness of the population in the interval (t_{k-1}, t_k). Unlike in the birth-death process, the variance parameter κ_k, which now characterizes all the noise in the BLT experiments including growth, dilution, DNA extraction, DNA amplification, etc., is unknown a priori. To determine κ_k, Levy et al performed additional experiments and estimated $\kappa_k \approx 3$ (see equations (40, 45) in the Supplementary Information to Ref. [2]). However, κ_k is treated as a fitting parameter when BLT data are analyzed, as described below.

Levy et al apply the following procedure to infer the lineage selection coefficients s from the BLT data. At each cycle k, they first infer the mean fitness $\bar{s}_k$ using low-abundance lineages, i.e. those with read counts $20 \le r_{k-1} \le 40$. The assumption is that these lineages are so small in size that they have not acquired any adaptive mutations, and so the selection coefficient s for such lineages is zero. For such lineages, equation (S9) simplifies to equation (1) in the main text, which can be used to infer μ_k and therefore $\bar{s}_k$. However, instead, Levy et al construct the entire empirical distribution for r_k and fit equations (S8), (S9) to it, using $\bar{s}_k$ and κ_k as free parameters (see equations (45)–(48) in the Supplementary Information to Ref. [2]).

Having inferred the $\bar{\boldsymbol{s}} = (\bar{s}_1, \bar{s}_2, \dots)$ and $\boldsymbol{\kappa} = (\kappa_1, \kappa_2, \dots)$ from low-abundance lineages, the LB approach takes the entire barcode read-count trajectory $\boldsymbol{r}_i = (r_{i1}, r_{i2}, \dots)$ for each lineage i to infer its selection coefficient s_i and the establishment time τ_i, using a Bayesian approach. Specifically, they define the likelihood

function for the read-count trajectory $\boldsymbol{r}_i$ as

$$L_i(s,\tau) = \prod_k p\big(r_{ik}; \mu_k(s, \bar{s}_k, \tau), \kappa_k\big), \tag{S10}$$

where p is given by equation (S8) and an expression for $\mu_k(s_i, \bar{s}_k, \tau_i)$ that is adjusted to account for the fact that an adapted lineage contains a subpopulation of adapted cells with $s_i > 0$ growing from established time τ_i and a non-adapted subpopulation (see equations (58)–(62) in the Supplementary Information to Ref. [2]). To determine if lineage i is adapted or not, they construct the ratio of posterior probabilities as

$$B_i(s,\tau) = \frac{P^{\text{prior}}(s_i, \tau_i) \times L_i(s_i, \tau_i)}{P^{\text{prior}}(\text{no mutation}) \times L_i(0,0)}, \tag{S11}$$

where P^{prior} denote prior probability for each hypothesis (see equations (64)–(67) in the Supplementary Information to Ref. [2]). Finally, they find values $\hat{s}_i$ and $\hat{\tau}_i$ that maximize B_i. Lineage i is identified as adapted if $B_i(\hat{s}_i, \hat{\tau}_i) > 1$.

2.2 Review of FitMut2

FitMut2 is an improved algorithm based on the LB method. The key difference between the LB method and FitMut2 is that FitMut2 uses an iterative approach to simultaneously call adapted lineages and infer mean fitness from them in a consistent way, which removes the need for a large number of neutral lineages required by the LB method. For convenience, we provide the summary of similarities and differences between all the BLT analysis methods used in this paper in Table A.

Specifically, for each lineage i, FitMut2 considers two hypotheses, that the lineage acquired an adaptive mutation ($\Theta = 1$) or not ($\Theta = 0$) during the BLT experiment. FitMut2 calculates the probability for the observed read-count trajectory $\boldsymbol{r}_i$ for lineage i as

$$p(\boldsymbol{r}_i) = p(\boldsymbol{r}_i \,|\, \Theta = 0)\, p(\Theta = 0) + p(\boldsymbol{r}_i \,|\, \Theta = 1)\, p(\Theta = 1), \tag{S12}$$

where $p\,(\Theta = 1)$ and $p\,(\Theta = 0)$ are the prior probabilities of the adaptive and neutral hypotheses, respectively.

For the neutral model ($\Theta = 0$), they set $p(\Theta = 0) = 1$ and expand the probability of the trajectory as

$$p(\boldsymbol{r}_i \,|\, \Theta = 0) = p(r_{i0} \,|\, \Theta = 0) \prod_j p(r_{ij} \,|\, r_{ij-1}, \Theta = 0),$$

where $p(r_{i0} \,|\Theta = 0) = p(r_{i0})$ is the initial prior (see equations (14)–(15) in the Supplementary Information to Ref. [3]) and the transition probability $p(r_{ij} \,|\, r_{ij-1}, \Theta = 0)$

	**Levy-Blundell (LB method)**	**FitMut2 (modified LB)**	**BASIL (this work)**
Evolution model	Branching process starting from a single cell	Same as the LB method	Deterministic exponential growth and stochastic dilution
Measurement model	Custom model with variance proportional to mean	Same as the LB method	Negative binomial with variance quadratic in mean
Lineage size n	Not modeled explicitly	Not modeled explicitly	Bayesian belief distribution (Sec. 3.2)
Read count r	Eq. (S8)	Eq. (S12)	Eq. (S47)
Estimation of mean fitness $\bar{s}$	Maximum likelihood for reference lineages	Direct calculation based on identified adapted lineages	Maximum likelihood for reference lineages
Reference lineages	All small lineages (assumed neutral)	Recursively select all putatively adapted lineages	Random lineages (both putatively neutral and/or adapted)
Neutral decline assumption	Yes	No	No
Lineage identification	Ratio of posterior probabilities (Eq. (S11))	Bayesian model choice (Eq. (S13))	Belief distribution for s (Sec. 3.4)
Fitness effect inference, s	Maximum likelihood for r trajectory (Eq. (S10))	Maximum likelihood for r trajectory	Mean of belief distribution for s (Sec. 3.4)

Table A. Summary of BLT methods.

is given by equation (S7)) with the expected read count μ_k given by equation (S9) with $s = 0$ (see equation (11) in the Supplementary Information to Ref. [3]).

For the adaptive model ($\Theta = 1$), they express the probability $p\,(\boldsymbol{r}_i \,|\, \Theta = 1)\; p\,(\Theta = 1)$ as an integral over all possible ways to be an adapted lineage with fitness effects s and establishment time τ

$$p(\boldsymbol{r}_i \,|\, \Theta = 1)\, p(\Theta = 1) = \int p(\boldsymbol{r}_i \,|\, s, \tau)\, p(s, \tau)\, ds\, d\tau,$$

where $p(s, \tau)$ is the prior probability that a lineage acquires a beneficial mutation (defined in the Discussion of Ref. [3]). Then the probability of the adaptive trajectory is factorized as

$$p(\boldsymbol{r}_i \,|\, s, \tau) = p(r_{i0} \,|\, s, \tau) \prod_j p(r_{ij} \,|\, r_{ij-1},\; s, \tau),$$

where $p(r_{i0} \,|\, s, \tau) = p(r_{i0})$ is the same initial prior as in the neutral model and $p(r_{ij} \,|\, r_{ij-1},\; s, \tau)$ is given by equation (S7) with the same expected read count μ_k as in the LB method (essentially equation (S9) adjusted for the fact that the adaptive lineage is segregating within the barcoded subpopulation), defined in equation (13) in the Supplementary Information to Ref. [3].

To choose the best model for lineage i, FitMut2 applies Bayes' theorem to calculate the posterior probability of $\Theta = 1$, given the data $\boldsymbol{r}_i$,

$$p(\Theta = 1 \,|\, \boldsymbol{r}_i) = \frac{p(\boldsymbol{r}_i \,|\, \Theta = 1)\, p(\Theta = 1)}{p(\boldsymbol{r}_i)}, \tag{S13}$$

where $p\,(\{r_{ik}\})$ is given by equation (S12). If $p\,(\Theta = 1 \,|\, \boldsymbol{r}_i) > 0.5$, lineage i is called putatively adaptive, with putative estimates of s and τ obtained by maximizing the posterior log-likelihood $L(s, \tau) = \ln\left(p(\boldsymbol{r}_i \,|\, s, \tau)\; p(s, \tau)\right)$.

Then, they calculate the mean fitness trajectory $\bar{\boldsymbol{s}}$ at time k from the frequencies f_{ik} of all putatively adapted lineages i

$$\bar{s}_k = \sum_i f_{ik}\, s_i. \tag{S14}$$

With the newly estimated mean fitness trajectory $\bar{\boldsymbol{s}}$, FitMut2 then re-identifies putatively adaptive lineages and re-estimates their selection coefficients and establishment times. FitMut2 then continues this process iteratively until it converges.

2.3 Key assumptions of the Levy-Blundell method

The LB method—and the neutral decline method as its simplified version—is based on two key assumptions. In this section, we discuss their validity and possible consequences for inference if they are violated.

2.3.1 Assumption 1. Low-abundance lineages are neutral

Low-abundance lineages can be used to infer the mean-fitness trajectory as long as these lineages are neutral, i.e., as long as they have not yet acquired adaptive mutations. If the lineages chosen as reference are in fact not neutral, the inferred mean fitness will be biased, as we demonstrate in the main text. Thus, it is critical for the accuracy of the LB method that this assumption holds.

Of course, as adaptation proceeds, all neutral lineages eventually go extinct and this assumption will eventually be violated. The time scale of persistence of neutral lineages depends on the speed of adaptation, which in turn is determined by the availability of adaptive mutations and their fitness effects, and is therefore a priori unknown.

This assumption can be relaxed if we allow for adapted lineages with known fitness to be used as reference. This can be implemented in practice as follows. By definition, in an initially clonal population, most cells are "wildtype" and any lineage can be used as reference at the initial phase of adaptation. Some lineages will acquire adaptive mutations earlier than others and their fitness can be estimated reliably while neutral lineages are still present in the population. These early adapted lineages can be then used as reference themselves until they acquire secondary adaptive mutations.

2.3.2 Assumption 2. Read frequency dynamics faithfully represent lineage frequency dynamics

According to the population genetics theory, the expected rate of change dx/dt in the frequency of a mutant is determined by its current frequency x, its selection coefficient s and population's mean fitness $\bar{s}$,

$$\frac{dx}{dt} = x(s - \bar{s}).$$

Taking the mean fitness as constant $\bar{s}_k$ over a short period of time $\Delta t_k = t_k - t_{k-1}$ (e.g., between two consecutive sampling time points t_{k-1} and t_k in the BLT experiment), we integrate this equation and obtain the expected frequency x_k at time t_k,

$$x_k = x_{k-1} e^{(s-\bar{s}_k)\Delta t_k}. \tag{S15}$$

Thus, if the lineage frequencies are known, we can solve equation (S15) for $\bar{s}_k$ (if s is known) or for s (if $\bar{s}_k$ is known).

However, x_k are never known with certainty but are estimated. In particular, in BLT experiments, x_k must be estimated from the corresponding barcode read-count data. The LB method (and the neutral decline method as a consequence) makes an implicit but crucial assumption that the dynamics of measured barcode

read frequencies are an unbiased (albeit noisy) representation of the dynamics of *cell* frequencies. While this assumption is reasonable, it is in general not true.

We will first show that this assumption is indeed implied by equation (S9). Since μ_k is the expected number of reads at time t_k, equation (S9) can also be re-written as

$$\frac{\mathbb{E}\left(r_k | r_{k-1}\right)}{R_k} = \frac{r_{k-1}}{R_{k-1}} e^{(s-\bar{s}_k)\Delta t_k}, \tag{S16}$$

which implies that the expected read frequency $\mathbb{E}\left(r_k|r_{k-1}\right)/R_k$ depends on the previous read frequency r_{k-1}/R_{k-1} in exactly the same way as the cell frequency x_k in equation (S15).

We will now show that equation (S16) is in fact not generally true. Suppose that the measurement process is described by the probability distribution $P^{\text{meas}}(r|x; R)$, which determines the number of reads r that are obtained from a lineage whose actual population frequency is x if the total read depth is R. Let us assume that measurement is unbiased, i.e.,

$$\mathbb{E}\left(r|x\right) = \sum_{r=1}^{R} r\, P^{\text{meas}}(r|x; R) = xR, \tag{S17}$$

which is supported by our data (see Figure 4A in the main text). We rewrite $\mathbb{E}\left(r_k|r_{k-1}\right)$ in terms of the measurement process P^{meas} and the evolutionary process P^{evol}.

$$\begin{aligned}
\mathbb{E}\left(r_k|r_{k-1}\right) &= \int_0^1 dx_{k-1} \int_0^1 dx_k\, P^{\text{evol}}(x_k|x_{k-1})\, P(x_{k-1}|r_{k-1}) \sum_{r_k} r_k P^{\text{meas}}(r_k|x_k) \\
&= R_k \int_0^1 dx_{k-1}\, P(x_{k-1}|r_{k-1}) \int_0^1 dx_k\, x_k\, P^{\text{evol}}(x_k|x_{k-1}) \\
&= R_k\, e^{(s-\bar{s}_k)\Delta t_k} \int_0^1 dx_{k-1}\, x_{k-1}\, P(x_{k-1}|r_{k-1}) \\
&= R_k\, e^{(s-\bar{s}_k)\Delta t_k}\, \mathbb{E}\left(x_{k-1}|r_{k-1}\right),
\end{aligned} \tag{S18}$$

where to simplify the notations we dropped the parameters from the expressions for various probability distributions. Equation (S18) shows that equation (S16) would be true if the expected frequency of lineages represented by r out of R reads is in fact r/R,

$$\mathbb{E}\left(x|r\right) = \frac{r}{R}. \tag{S19}$$

Counterintuitively, equation (S19) is in general not true. To demonstrate this, we first use the Bayes' theorem to re-write it as

$$\mathbb{E}\left(x|r\right) = \int_0^1 x\, P(x|r)\, dx = \int_0^1 x\, \frac{P^{\text{meas}}(r|x)\, P^{\text{prior}}(x)}{P(r)}\, dx. \tag{S20}$$

Here, $P(r)$ is the normalization constant and $P^{\text{prior}}(x)$ is the a priori probability that the frequency of the focal lineage in the population is x. Note that in general $P^{\text{prior}}(x)$ is not the distribution of lineage frequencies in the population. Indeed, at later time points in the BLT experiment, $P^{\text{prior}}(x)$ for different lineages are different, because they are informed by previous observations. However, at the beginning of the BLT experiment, all lineages are a priori identical, and $P^{\text{prior}}(x)$ can be approximated by the distribution of lineage frequencies in the population. Previous BLT studies have shown that, despite efforts to introduce all barcodes at the same frequency, the initial distributions of barcode frequencies are often be quite broad and may be close to exponential [1]. We now use four specific examples to show that equation (S19) does not hold.

Example 1. Poisson measurement noise and a uniform frequency distribution. First, we consider the simplest case, when the prior frequency distribution is uniform and the measurement noise is Poisson, i.e.,

$$\begin{aligned} P^{\text{prior}}(x)\,dx &= dx, \\ P^{\text{meas}}(r|x;R) &= \frac{(xR)^r\,e^{-xR}}{r!}. \end{aligned} \tag{S21}$$

We show in Appendix B, that the conditional distribution for frequency x given the read count r is approximately a gamma distribution with shape parameter $r+1$ and scale parameter $1/R$, such that

$$\mathbb{E}\left(x|r\right) \approx \frac{r+1}{R}. \tag{S22}$$

Reassuringly, when $r \gg 1$, expression (S22) converges to r/R, suggesting that Assumption 2 is reasonably accurate as long as all lineages are represented by many reads. However, at small numbers of reads, expression (S22) can deviate substantially from equation (S19).

Example 2. Poisson measurement noise and an exponential frequency distribution. We now consider an exponential frequency distribution, which may be adequate for initial time points [1],

$$P^{\text{prior}}(x)\,dx = N_L e^{-N_L x}\,dx, \tag{S23}$$

where N_L is the number of barcoded lineages, such that $\mathbb{E}\left(x\right) = 1/N_L$. We show in Appendix B, that the conditional distribution for frequency x given the read count r is approximately a gamma distribution with shape parameter $r+1$ and scale parameter $1/(R+N_L)$, such that

$$\mathbb{E}\left(x|r\right) \approx \frac{r+1}{R+N_L}. \tag{S24}$$

Since $R/N_L \gtrsim 100$ in typical BLT experiments, expression (S24) is still close to r/R for large r, but it again can deviate substantially from (S19) when the number of reads is small.

Example 3. Measurement noise with an increasing variance to mean ratio and a Gamma frequency distribution. We now consider a more realistic noise model, where the variance to the mean ratio increases with the mean according to equation (2) in the main text. We model the read count as a negative binomial random variable with mean xR and the variance to the mean ratio $1 + \epsilon xR$.

$$P^{\text{meas}}(r|x; R, \epsilon) = \frac{\Gamma(r + 1/\epsilon)}{\Gamma(r+1)\,\Gamma(1/\epsilon)}(1-p)^r\, p^{1/\epsilon}. \tag{S25}$$

with $p = (1+\epsilon xR)^{-1}$. We also consider a Gamma frequency distribution with shape parameter α and scale parameter $1/\tilde{N}$,

$$P^{\text{prior}}(x) = \frac{\tilde{N}^\alpha}{\Gamma(\alpha)} x^{\alpha-1} e^{-\tilde{N}x}, \tag{S26}$$

which may be a more accurate description of our prior knowledge at later time points with $\alpha/\tilde{N}$ representing the a priori expected frequency. If $\epsilon \ll 1$ and $r \ll 1/\epsilon$, the posterior distribution is approximately Gamma with shape parameter $r + \alpha$ and scale parameter $(R + \tilde{N})^{-1}$ (see Appendix B), such that

$$\mathbb{E}\,(x|r) = \frac{r+\alpha}{R+\tilde{N}}. \tag{S27}$$

Example 4. Measurement noise with a constant variance to mean ratio and an exponential frequency distribution. Finally, we consider a noise model where the variance to the mean ratio is constant and equal to 2κ as in Ref. [2]. We again model the read count as a negative binomial random variable,

$$P^{\text{meas}}(r|x) = \frac{\Gamma(r+k)}{\Gamma(r+1)\Gamma(k)}(1-p)^r p^k.$$

where $p = 1/2\kappa$ and $k = xR/(2\kappa - 1)$. In this case, as we show in Appendix B, we have

$$\mathbb{E}\,(x|r) \approx \frac{2\kappa + r}{R}, \quad r = 0, 1, \ldots, R. \tag{S28}$$

Substituting any of the equations (S22), (S24), (S27) or (S28) into equation (S18), we see that the relationship between the expected number of reads at time t_k and the observed number of reads at t_{k-1} is not the same as the relationship (S15) between the expected lineage frequency at time t_k and the actual frequency at t_{k-1}.

Specifically, unlike the relationship between $\mathbb{E}\left(x_k|x_{k-1}\right)$ and x_{k-1}, the relationship between $\mathbb{E}\left(r_k|r_{k-1}\right)$ and r_{k-1} appears to generally have a non-zero y-intercept, whose value depends on the parameters of the prior distribution and measurement noise. However, these two relationships become identical when r_{k-1} is sufficiently large, although how large it needs to be depends on the noise model.

3 BASIL

Notation used in this section are provided in Table B.

3.1 Model of measurement

As discussed in the main text, we cannot directly observe the size n of a barcoded lineage. Instead, a sample from the population is sequenced, and we observe the number of reads that contain the focal barcode.

We model the measurement process with the negative binomial distribution with mean $\langle r\rangle = nR/N$ and variance $\sigma_r^2 = \langle r\rangle + \epsilon \langle r\rangle^2$ (see Section 1), where N is total number of cells in the population during sampling, R is the total coverage, and r is the number of reads that contain the focal barcode, and ϵ is a free parameter controlling overdispersion. The negative binomial distribution is typically parameterized by the number of "successes" k and the success probability p, with mean and variance expressed in terms of these parameters as

$$\langle r\rangle = \frac{k(1-p)}{p}$$
$$\sigma_r^2 = \frac{k(1-p)}{p^2} = \frac{\langle r\rangle}{p},$$

which yield

$$k = \frac{p}{1-p}\langle r\rangle = \frac{1}{\epsilon}, \tag{S29}$$
$$p = \frac{1}{1+\epsilon\langle r\rangle}.$$

Thus, the probability of observing r reads for lineage with n cells is

$$P^{\mathrm{meas}}(r|n;\epsilon) = \frac{\Gamma\left(r+1/\epsilon\right)}{\Gamma\left(r+1\right)\Gamma\left(1/\epsilon\right)}\left(\frac{\epsilon\langle r\rangle}{1+\epsilon\langle r\rangle}\right)^r\left(\frac{1}{1+\epsilon\langle r\rangle}\right)^{\frac{1}{\epsilon}} \tag{S30}$$

with $\langle r\rangle = nR/N$. The measurement distribution (S30) depends on the noise parameter ϵ, which we fit, as well as on N and R which are assumed to be known. Furthermore, this distribution becomes a point measure at $r = 0$ if $n = 0$ and it converges to the Poisson distribution with mean $\langle r\rangle = nR/N$ as $\epsilon \to 0$.

Notation	Description	Eqns
k	sampling time index, $k \in [0, k_\text{f}]$	
t_k	time (in generation) of the kth sample	
R_k	total read depth at t_k	
D	dilution ratio, $D > 1$	
N	population size before dilution	
N_b	population size after dilution, $N_b = N/D$	
ϵ	measurement error parameter	(2)
s_i	selection coefficient (fitness) of the ith lineage	
$\bar{s}_k$	mean fitness of the population in time interval (t_{k-1}, t_k) for all $k \geq 1$	
n_{ik}	size of ith lineage at t_k before dilution	
r_{ik}	barcode read count of ith lineage at t_k	
$\boldsymbol{r}_{ik}$	observation vector for lineage i prior to and including t_k, $\boldsymbol{r}_{ik} = (r_{i0}, \ldots, r_{ik})$	
$P^\text{belief}(n, s\|\boldsymbol{r})$	belief probability that a lineage with the observation vector $\boldsymbol{r}$ has size n and fitness s	(3), (S46)
$P^\text{belief}_{ik}(n, s)$	shorthand for $P^\text{belief}(n, s\|\boldsymbol{r}_{ik})$	
$P^\text{meas}(r\|n)$	probability of observing r reads for a lineage with size n. P^meas depends on the total read depth R and the measurement error parameter ϵ	(S30)
$P^\text{prior}(n, s\|\boldsymbol{r}')$	prior probability that a lineage with the observation vector $\boldsymbol{r}'$ has size n and selection coefficient s at the *next* sampling time point. P^prior depends on the current mean fitness $\bar{s}$	(S44)
$Z^\text{neut}(r_{ik})$	marginal likelihood of r_{ik} in the neutral model. Z^neut depends on the current mean fitness $\bar{s}$ and measurement error parameter ϵ	(S66)
$Z^\text{sel}(r_{ik})$	marginal likelihood of r_{ik} in the selection model. Z^sel depends on the current mean fitness $\bar{s}$ and measurement error parameter ϵ	(S47)
$L_k(\bar{s}, \epsilon)$	log-likelihood function for the mean fitness $\bar{s}$ and measurement error parameter ϵ	(S63)

Table B. Notations used in BASIL.

3.2 The belief distribution for lineage size and fitness

Our main object is the belief probability distribution $P^{\text{belief}}(n, s \,|\, \boldsymbol{r})$ that a lineage with the observation vector $\boldsymbol{r} = (r_0, \ldots, r_k)$ has size n and selection coefficient s. For a given lineage i whose observation vector up to and including time point t_k is $\boldsymbol{r}_{ik} = (r_{i0}, \ldots, r_{ik})$, we will sometimes use a simplified notation $P^{\text{belief}}_{ik}(n, s)$ instead of $P^{\text{belief}}(n, s \,|\, \boldsymbol{r}_{ik})$. For mathematical tractability, we express the belief distribution as

$$P^{\text{belief}}(n, s|\boldsymbol{r}) = P^{\text{belief}}(n|s, \boldsymbol{r})\, P^{\text{belief}}(s|\boldsymbol{r}) \tag{S31}$$

and assume the following parametric forms

$$P^{\text{belief}}(n|s, \boldsymbol{r}) = \gamma\left(n\,;\, \varkappa(s, \boldsymbol{r}), \theta(s, \boldsymbol{r})\right), \tag{S32}$$

$$P^{\text{belief}}(s|\boldsymbol{r}) = N\left(s\,;\, \mu(\boldsymbol{r}), \sigma^2(\boldsymbol{r})\right), \tag{S33}$$

where $N(s; \mu, \sigma^2)$ is the normal distribution with mean μ and variance σ^2 and $\gamma\,(n; \varkappa, \theta)$ is the gamma distribution with shape parameter $\varkappa$ and scale parameter θ.

Thus, $P^{\text{belief}}(n, s|\boldsymbol{r})$ belongs to a family of distributions with four parameters μ, σ^2, $\varkappa$, θ where the latter two parameters can be functions of s. In this section, we describe how we update these parameters as the observation vector is augmented from one time point to the next. We do so in two steps, which we refer to as "projection" and "update". During the projection step, which is described in Section 3.2.1, we project the past belief distribution P^{belief}_{ik-1} to obtain the prior distribution P^{prior}_{ik} for the time point t_k. During the update step, which is described in Section 3.2.2, we use the Bayes' theorem to obtain the new belief distribution P^{belief}_{ik} based on the prior distribution P^{prior}_{ik} and the read count r_{ik} observed at time t_k (see equation (3) in the main text). Importantly, the parametric distribution given by equation (S32) has no weight at $n = 0$. In other words, it is valid only for lineages that are present in the population and have not yet gone extinct. We describe how we treat lineage extinctions in Section 3.2.2.

3.2.1 Projecting the belief distribution

To obtain the prior distribution $P^{\text{prior}}(n, s|\boldsymbol{r}')$ for the time point t_k, where $\boldsymbol{r}' = (r_0, \ldots, r_{k-1})$ is the observation vector up to and including the previous time point t_{k-1}, we assume that the belief distribution $P^{\text{belief}}(n, s|\boldsymbol{r}')$ at the previous time point is known and has the parametric form given by equations (S32), (S33) with parameters μ, σ, θ, $\varkappa$. To simplify notations, in this section, we will omit the explicit dependence of all probabilities on the observation vector $\boldsymbol{r}'$.

The sampling time points $t_0, t_1, \ldots$ in the BLT experiment may be separated by one or multiple growth and dilution cycles. We will first consider one such cycle and

obtain the "1-cycle projected distribution" $P_1(n,s)$ whose parameters we denote as μ_1, σ_1, $\varkappa_1$, and θ_1. We then use $P_1(n,s)$ to obtain the prior probability for n and s after multiple cycles.

To derive $P_1(n,s)$, we consider a barcoded lineage that has fitness s relative to the ancestor and that is represented by n cells immediately prior to dilution. At dilution, a fraction $1/D$ of the population is transferred into fresh medium, and the rest is discarded. Immediately after dilution, the lineage's size becomes n', which we model as a Poisson random variable with mean n/D, such that

$$P^{\mathrm{dil}}(n') = \frac{\left(\frac{n}{D}\right)^{n'} e^{-n/D}}{\Gamma(n'+1)}, \quad n' = 0, 1, \ldots$$

We assume that after dilution, the lineage grows deterministically based on the difference between its fitness s and the population's mean fitness $\bar{s}$, such that by the end of the cycle, its size is $n_1 = An'$, where

$$A = De^{(s-\bar{s})\Delta t_c}$$

and Δt_c is the cycle duration in generations. Then, the one-cycle transition probability is

$$P^{\mathrm{c}}(n_1 \mid n; s, \bar{s}) = P^{\mathrm{dil}}\left(\frac{n_1}{A}\right) = \frac{\left(\frac{n}{D}\right)^{n_1/A} e^{-n/D}}{\Gamma\left(\frac{n_1}{A}+1\right)}, \tag{S34}$$

where formally $n_1/A = 0, 1, 2, \ldots$. Then, for the 1-cycle projected distribution we have

$$P_1(n_1, s) = \int_0^\infty P^{\mathrm{c}}(n_1 \mid n; s, \bar{s})\; P^{\mathrm{belief}}(n,s)\; dn. \tag{S35}$$

Integrating equation (S35) with respect to n_1, we find, as expected, that the projection does not change the marginal distribution for s,

$$P_1(s) = P^{\mathrm{belief}}(s) = N\left(s\,;\,\mu, \sigma^2\right),$$

which implies that $\mu_1 = \mu$ and $\sigma_1 = \sigma$. Since we can express the joint distribution $P_1(n_1, s)$ through this marginal distribution as $P_1(n_1, s) = P_1(n_1 \mid s)\; P_1(s)$, we now only need to obtain the conditional probability $P_1(n_1 \mid s)$. To do so, we note that

$$\begin{aligned} P_1(n_1 \mid s) = \frac{P_1(n_1, s)}{P_1(s)} &= \frac{\int_0^\infty dn\, P^{\mathrm{c}}(n_1 \mid n; s, \bar{s}_k)\; P^{\mathrm{belief}}(n \mid s)\, P^{\mathrm{belief}}(s)}{P^{\mathrm{belief}}(s)} \\ &= \int_0^\infty P^{\mathrm{c}}(n_1 \mid n; s, \bar{s}_k)\; P^{\mathrm{belief}}(n \mid s)\, dn. \end{aligned} \tag{S36}$$

Substituting expressions (S34) and (S32) into equation (S36), we obtain

$$
\begin{aligned}
P_1\left(n_1 \,|\, s\right) &= \frac{1}{D^{\frac{n_1}{A}}\,\Gamma\left(\frac{n_1}{A}+1\right)\,\Gamma(\varkappa)\theta^{\varkappa}} \int_0^{\infty} n^{\tilde{\varkappa}-1} e^{-\frac{n}{\tilde{\theta}}}\, dn \\
&= \frac{\Gamma\left(\frac{n_1}{A}+\varkappa\right)}{\Gamma\left(\frac{n_1}{A}+1\right)\,\Gamma(\varkappa)} \left(\frac{\theta}{D+\theta}\right)^{\frac{n_1}{A}} \left(\frac{D}{D+\theta}\right)^{\varkappa}, \qquad \text{(S37)}
\end{aligned}
$$

with $n_1/A = 0, 1, 2, \ldots$, $\tilde{\varkappa} = \frac{n_1}{A} + \varkappa$, $\tilde{\theta} = \frac{\theta D}{\theta + D}$. Equation (S37) shows that, conditional on s, n_1/A is distributed as negative binomial with success probability $D/(D+\theta)$ and number of successes $\varkappa$. Therefore,

$$
\begin{aligned}
\mathbb{E}\left(n_1|s\right) &= \varkappa\,\theta\, e^{(s-\bar{s})\Delta t_c}, \\
\mathrm{Var}\left(n_1|s\right) &= \varkappa\,\theta\,(D+\theta) e^{2(s-\bar{s})\Delta t_c}.
\end{aligned}
$$

Furthermore, the lineage goes extinct after one growth and dilution cycle with probability

$$
p_{\mathrm{ext},1}(\theta,\varkappa) = \left(\frac{D}{D+\theta}\right)^{\varkappa} \qquad \text{(S38)}
$$

and survives with probability

$$
p_{\mathrm{surv},1}(\theta,\varkappa) = 1 - \left(\frac{D}{D+\theta}\right)^{\varkappa}. \qquad \text{(S39)}
$$

It is then easy to show that the mean and variance conditional on survival are

$$
\begin{aligned}
\mathbb{E}\left(n_1 \,|\, s, \mathrm{surv}\right) &= \frac{\varkappa\,\theta}{p_{\mathrm{surv},1}(\theta,\varkappa)}\, e^{(s-\bar{s})\Delta t_c}, \qquad \text{(S40)} \\
\mathrm{Var}\left(n_1 \,|\, s, \mathrm{surv}\right) &= \frac{p_{\mathrm{surv},1}(\theta,\varkappa)\,(D+\theta+\varkappa\,\theta) - \varkappa\,\theta}{p^2_{\mathrm{surv},1}(\theta,\varkappa)} \varkappa\,\theta\, e^{2(s-\bar{s})\Delta t_c}. \qquad \text{(S41)}
\end{aligned}
$$

To facilitate further calculations, we approximate the distribution of n_1, conditional on s and lineage survival, by a gamma distribution with the shape parameter $\varkappa_1$ and scale parameter θ_1. To determine $\varkappa_1$ and θ_1, we equate the mean $\varkappa_1\theta_1$ and variance $\varkappa_1\theta_1^2$ of this gamma distribution to the conditional mean and variances given by equations (S40) and (S41) and find $\theta_1 = f_\theta(s,\theta,\varkappa)$ and $\varkappa_1 = f_\varkappa(\theta,\varkappa)$ where

$$
\begin{aligned}
f_\theta(s,\theta,\varkappa) &= \frac{p_{\mathrm{surv},1}(\theta,\varkappa)\,(\theta + D + \varkappa\theta) - \varkappa\theta}{p_{\mathrm{surv},1}(\theta,\varkappa)}\, e^{(s-\bar{s}_k)\Delta t_c}, \qquad \text{(S42)} \\
f_\varkappa(\theta,\varkappa) &= \frac{\varkappa\theta}{p_{\mathrm{surv},1}(\theta,\varkappa)\,(\theta + D + \varkappa\theta) - \varkappa\theta}. \qquad \text{(S43)}
\end{aligned}
$$

Therefore, the full projected probability for a lineage after one growth and dilution cycle is

$$P_1(n,s) = N\left(s;\mu,\sigma\right) \times \begin{cases} p_{\mathrm{ext},1}(\theta,\varkappa)\,, & \text{if } n=0, \\ p_{\mathrm{surv},1}(\theta,\varkappa)\,\gamma\left(n;\varkappa_1,\theta_1\right)\,, & \text{if } n>0, \end{cases}$$

where $p_{\mathrm{ext},1}$ and $p_{\mathrm{surv},1}$ are given by equations (S38) and (S39) and parameters θ_1 and $\varkappa_1$ are the functions of θ and $\varkappa$ given by equations (S42) and (S43).

If the next sampling occurs at the end of a single growth and dilution cycle after the previous sampling, then we set $P^{\mathrm{prior}}(n,s) = P_1(n,s)$. If $L>1$ growth and dilution cycles elapse between successive samples, then we set the prior probability $P^{\mathrm{prior}}(n,s)$ for the next sampling time point to be equal to

$$P^{\mathrm{prior}}(n,s) = N\left(s;\mu,\sigma\right) \times \begin{cases} 1-p_{\mathrm{surv},L}\,, & \text{if } n=0, \\ p_{\mathrm{surv},L}\,\gamma\left(n;\varkappa_L,\theta_L\right)\,, & \text{if } n>0, \end{cases} \tag{S44}$$

where the L-cycle survival probability is given by

$$p_{\mathrm{surv},L} = \prod_{\ell=1}^{L} p_{\mathrm{surv},1}(\theta_\ell,\varkappa_\ell), \tag{S45}$$

and parameters $\theta_\ell = f_\theta\left(s,\theta_{\ell-1},\varkappa_{\ell-1}\right)$, $\varkappa_\ell = f_\varkappa\left(\theta_{\ell-1},\varkappa_{\ell-1}\right)$ for $\ell = 1,2,\ldots L$ are obtained recursively using equations (S42) and (S43) with $\varkappa_0 \equiv \varkappa$, $\theta_0 \equiv \theta$. Note that since all $\varkappa_\ell$ and θ_ℓ depend on the parameters $\varkappa$, θ of the prior belief distribution $P^{\mathrm{belief}}(n\,|\,s,\boldsymbol{r}')$, which themselves are functions of s, the survival probability $p_{\mathrm{surv},L}$ is also a function of s.

3.2.2 Updating the belief distribution

Next, we apply the Bayes' theorem to obtain the belief distribution $P^{\mathrm{belief}}\left(n,s\,|\,\boldsymbol{r}\right)$ after observing the current read count r_k,

$$P^{\mathrm{belief}}\left(n,s\,|\,\boldsymbol{r}\right) = \frac{P^{\mathrm{prior}}\left(n,s\,|\,\boldsymbol{r}'\right)\,P^{\mathrm{meas}}\left(r_k\,|\,n\right)}{Z^{\mathrm{sel}}(\boldsymbol{r})}, \tag{S46}$$

where $\boldsymbol{r}' = (r_0,\ldots,r_{k-1})$ and $\boldsymbol{r} = (r_0,\ldots,r_{k-1},r_k)$ are the previous and current observation vectors, $P^{\mathrm{prior}}\left(n,s\,|\,\boldsymbol{r}'\right)$ is the prior probability that the lineage has selection coefficient s and size n at the current observation time point before the current measurement is made (see equation (S44)), $P^{\mathrm{meas}}\left(r\,|\,n\right)$ is the probability of observing r reads for a lineage of size n (see equation (S30)), and the denominator

$$Z^{\mathrm{sel}}(\boldsymbol{r}) = \int_{-\infty}^{\infty} ds \int_0^{\infty} dn\, P^{\mathrm{prior}}\left(n,s\,|\,\boldsymbol{r}'\right)\,P^{\mathrm{meas}}\left(r_k\,|\,n\right). \tag{S47}$$

is the marginal probability of observing r_k reads in our model. The superscript "sel" stands for "selection" and denotes the fact that our model allows the selection coefficient of the lineage to vary freely. We will later consider a neutral null model where the selection coefficient is fixed at zero (see Section 3.3). Note that P^{prior} depends on $\bar{s}_k$, the mean fitness at the current interval (t_{k-1}, t_k), and P^{meas} depends on the noise parameters ϵ_k at the current interval (t_{k-1}, t_k). Therefore, both Z^{sel} and P^{belief} depend on both $\bar{s}_k$ and ϵ_k. For now, we can ignore these dependencies treating these parameters as fixed and known. However, these dependencies will becomes important in Section 3.3, where we discuss how $\bar{s}_k$ and ϵ_k are estimated.

As discussed above, we would like to approximate the belief distribution with an analytical parametric form given by equations (S32), (S33). However, since the numerator of equation (S46) is a complex function of both n and s, the normalization constant (S47) and the moments of this distribution cannot be expressed analytically. Therefore, to estimate the parameters of the belief distribution, we draw a random sample (n_j, s_j), $j = 1, 2, \ldots, M$ from the belief distribution $P^{\text{belief}}(n, s \,|\, \boldsymbol{r})$ using the Markov Chain Monte Carlo (MCMC) approach (see Section 3.5 for the details of the algorithm). We consider two cases, $r_k > 0$ and $r_k = 0$, which differ qualitatively because in the former case, the lineage is guaranteed to have survived until the current sampling time point t_k whereas in the later case it is possible that the lineage has gone extinct.

Guaranteed lineage survival when $r_k > 0$. If $r_k > 0$, that is, if the lineage is observed at current time point t_k, then $P^{\text{belief}}(0, s \,|\, \boldsymbol{r}) = 0$ since $P^{\text{meas}}(r_k \,|\, 0) = 0$ whenever $r_k > 0$. In other words, if we observe the lineage, we know with certainty that it has survived up to the current sampling time point. We then estimate the parameters μ, σ of the distribution (S33) from this sample as

$$\hat{\mu} = \frac{1}{M} \sum_{j=1}^{M} s_j, \tag{S48}$$

$$\hat{\sigma} = \sqrt{\frac{1}{M-1} \sum_{j=1}^{M} (s_j - \hat{\mu})^2}. \tag{S49}$$

Estimating parameters $\varkappa(s)$ and $\theta(s)$ of the conditional distribution (S32) is more difficult because they can be arbitrary functions of s. In principle, one could estimate these functions by independently fitting the shape and scale parameters of the gamma distribution for each s-slice of the joint distribution. However, this approach is computationally intensive. To reduce computational burden, we sought to find simple functional forms for the functions $\varkappa(s)$ and $\theta(s)$ and fitting the parameters of these functional forms directly from the joint distribution. To this end, we no-

ticed that $\ln n$ and s are linearly correlated in our MCMC samples (see Figure A), implying that

$$\mathbb{E}\left(\ln n|s\right) \approx \tilde{a} + \tilde{b}\, s. \tag{S50}$$

Since

$$\mathbb{E}\left(\ln n|s\right) = \psi(\varkappa) + \ln\theta \approx \ln\varkappa + \ln\theta,$$

where ψ is the di-gamma function (see Appendix C) and the approximation $\psi(\varkappa) \approx \ln\varkappa$ holds when $\varkappa \gg 1$, we obtain

$$\theta \approx \frac{e^{\tilde{a}+\tilde{b}s}}{\varkappa},$$

which suggests that an exponential dependence of θ on s should capture the shape of our belief distribution. To simplify subsequent calculations, we use the ansatz

$$\theta\left(s\right) = \frac{a}{\varkappa}\exp\left[\frac{b\left(s-\mu\right)}{\sigma}\right], \tag{S51}$$

where $\varkappa > 0$, a and b are the new real-valued parameters of distribution (S32).

With this parametrization, we have

$$\mathbb{E}\left(n\right) = a\, e^{\frac{b^2}{2}}, \tag{S52}$$

$$\mathrm{Var}\left(n\right) = \frac{a^2 e^{b^2}}{\varkappa}\left((1+\varkappa)e^{b^2} - \varkappa\right), \tag{S53}$$

$$\mathrm{Cov}\left(n, s\right) = ab\sigma\, e^{\frac{b^2}{2}}. \tag{S54}$$

Equations (S52)–(S54) can be solved to yield estimators of the parameters of the gamma distribution (S32) with the functional form (S51),

$$\hat{b} = \frac{\widehat{\mathrm{Cov}}\left(s, n\right)}{\langle n\rangle\ \hat{\sigma}} \tag{S55}$$

$$\hat{a} = \langle n\rangle\, e^{-\hat{b}^2/2} \tag{S56}$$

$$\hat{\varkappa} = \frac{\hat{a}^2 e^{2\hat{b}^2}}{\widehat{\mathrm{Var}}(n) - \hat{a}^2 e^{\hat{b}^2}(e^{\hat{b}^2} - 1)}, \tag{S57}$$

where

$$\langle n \rangle = \frac{1}{M} \sum_{j=1}^{M} n_j, \tag{S58}$$

$$\widehat{\mathrm{Var}}(n) = \frac{1}{M-1} \sum_{j=1}^{M} (n_j - \langle n \rangle)^2, \tag{S59}$$

$$\widehat{\mathrm{Cov}}(s, n) = \frac{1}{M-1} \sum_{j=1}^{M} (s_j - \hat{\mu})(n_j - \langle n \rangle). \tag{S60}$$

To test how well our parametric form (S32), (S33), (S51) with the parameters fitted using equations (S55)–(S60) fits the MCMC-sampled posterior distribution, we selected four lineages from simulated and real data and then plotted their MCMC-sampled marginal distributions for s and n as well as their joint distribution (Figure A). To calculate the marginal distribution for n, we generated $M' = 10^5$ random samples of s_j, $j = 1, \ldots, M'$ from the normal distribution with mean $\hat{\mu}$ and variance $\hat{\sigma}^2$. We then approximate the marginal distribution for n as

$$P^{\text{belief}}(n|\boldsymbol{r}) \approx \frac{1}{M'} \sum_{j=1}^{M'} \gamma\left(n\,;\,\hat{\varkappa}, \hat{\theta}_j\right), \tag{S61}$$

where $\hat{\theta}_j = \hat{a}/\hat{\varkappa} \exp\left(\hat{b}\,(s_j - \hat{\mu})/\hat{\sigma}\right)$ with s. Figure A shows that our parametric form captures the MCMC-sampled distribution reasonably well.

Possible lineage extinction when $r_k = 0$. If $r_k = 0$, there are two possibilities. With probability $p_{\text{surv},L}$ given by equation (S45), the lineage has survived up to the current sampling time point t_k but was not detected due to sampling noise; or, with probability $1 - p_{\text{surv},L}$, the lineage has gone extinct between t_{k-1} and t_k. Therefore, the belief distribution $P^{\text{belief}}(n, s|\boldsymbol{r})$ has a non-zero weight at $n = 0$,

$$P^{\text{belief}}(n, s) = \frac{N(s; \mu, \sigma)}{Z^{\text{sel}}} \times \begin{cases} 1 - p_{\text{surv},L}\,, & \text{if } n = 0, \\ p_{\text{surv},L}\, \frac{\gamma(n; \varkappa_L, \theta_L)}{(1+\epsilon_k nR/N)^{1/\epsilon_k}}, & \text{if } n > 0, \end{cases} \tag{S62}$$

with

$$\begin{aligned} Z^{\text{sel}} &= \int_{-\infty}^{\infty} N(s; \mu, \sigma) \left(1 - p_{\text{surv},L} + p_{\text{surv},L} \int_0^{\infty} \frac{\gamma\,(n; \varkappa_L, \theta_L)\; dn}{(1 + \epsilon_k nR/N)^{1/\epsilon_k}},\right) ds \\ &\approx \int_{-\infty}^{\infty} N(s; \mu, \sigma) \left(1 - p_{\text{surv},L} + p_{\text{surv},L} \left(\frac{R}{N\theta_L + R}\right)^{\varkappa_L}\right) ds, \end{aligned}$$

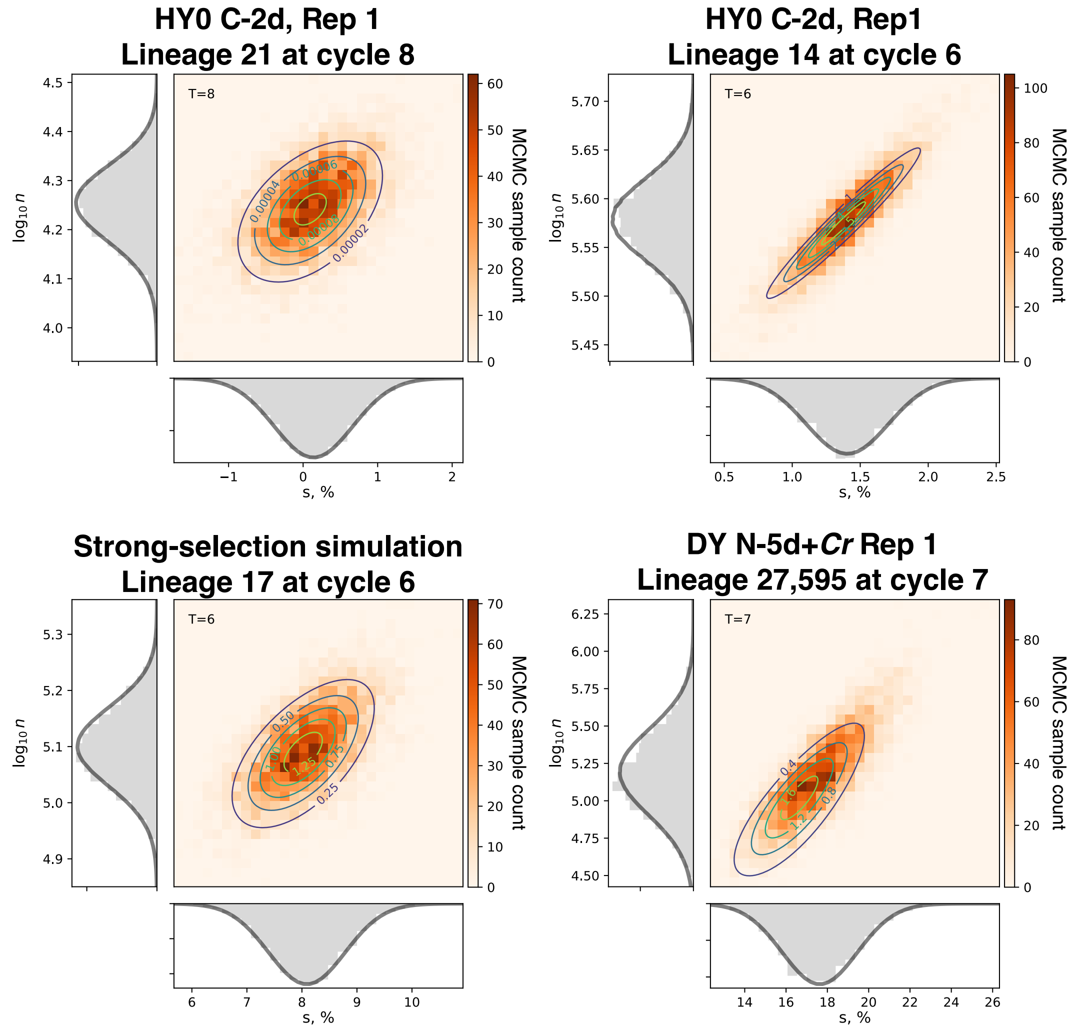

Figure A. Estimation of the belief distribution $P^{\mathrm{b}}(n, s)$. Each panel corresponds to a single lineage in a particular dataset at a certain time point, as indicated in the panel title. The heatmap in each panel shows the number of MCMC samples in each $(s, \log_{10} n)$ bin. The contour lines correspond to the analytical function $P^{\mathrm{b}}(n, s)$ given by equations (S31)–(S33) with parameters fitted to the MCMC sample. Histograms show the marginal distributions for s (bottom) and $\log_{10} n$ (left) and the corresponding parametric curves. See Section 3.2.2 for details.

where the approximation holds when $\epsilon_k \ll 1$, and we have omitted the dependencies on the observation vector $\boldsymbol{r}$ and mean fitness $\bar{s}_k$.

Equation (S62) shows that the belief distribution can no longer be adequately captured by the parametric form given by equations (S32), (S33). Instead, we use this parametric form to capture the conditional belief distribution, conditional on the lineage having survived until the current sampling time point t_k. We do so using the same approach as above, i.e., we obtain an MCMC sample (n_j, s_j), $j = 1, 2, \ldots, M$ from the distribution (S62) and then use equations (S58)–(S60) to estimate the mean and the variance of the lineage size n and the covariance between lineage size and selection coefficient, except we use only those MCMC samples with $n_j > 0$. We then apply equations (S48), (S49), (S55)–(S57) to estimate the parameters of the conditional belief distribution (S32), (S33). Furthermore, if the lineage was not observed at two consecutive time points, we assume that it has gone extinct and stop tracking it.

3.3 Estimating population's mean fitness and the noise parameter

As mentioned above, our model of evolution and consequently our procedure for updating the belief distribution at the "current" time point t_k depends on the population's mean fitness $\bar{s}_k$ during the interval (t_{k-1}, t_k), $k \geq 1$ as a parameter (see equation (S34)). Similarly, our measurement distribution (equation (S30)) depends on the noise parameter ϵ_k during the interval (t_{k-1}, t_k). So far, we have assumed that both $\bar{s}_k$ and ϵ_k are fixed and known. However, in reality the true values of $\bar{s}_k$ and ϵ_k are of course unknown and must be estimated before we update the lineage belief distributions at t_k.

We estimate $\bar{s}_k$ and ϵ_k using the maximum likelihood approach. To this end, we choose 3000 random lineages for which $r_{ik-1} > 0$ and classify each of them as either putatively neutral or putatively adapted (at the current time interval (t_{k-1}, t_k)) as described below. We denote the subsets of currently putatively neutral and putatively adapted lineages as $\mathcal{N}_k$ and $\mathcal{A}_k$, respectively. For each putatively adapted lineage $i \in \mathcal{A}_k$, we can treat the normalization constant $Z^{\mathrm{sel}}(\boldsymbol{r}_{ik}; \bar{s}_k, \epsilon_k)$ given by equation (S47) as the likelihood of observing the number of reads r_{ik} for that lineage at time t_k under the model with selection. Similarly, for each putatively neutral lineage $i \in \mathcal{N}_k$, we can treat the normalization constant $Z^{\mathrm{neut}}(\boldsymbol{r}_{ik}; \bar{s}_k, \epsilon_k)$ under the neutral model described below (see equation (S66)) as the likelihood of observing the number of reads r_{ik} for that lineage at time t_k under the neutral model. The procedures for estimating $Z^{\mathrm{sel}}(\boldsymbol{r}_{ik}; \bar{s}_k, \epsilon_k)$ and $Z^{\mathrm{neut}}(\boldsymbol{r}_{ik}; \bar{s}_k, \epsilon_k)$ are described below. Since both $Z^{\mathrm{sel}}(\boldsymbol{r}_{ik}; \bar{s}_k, \epsilon_k)$ and $Z^{\mathrm{neut}}(\boldsymbol{r}_{ik}; \bar{s}_k, \epsilon_k)$ depend on the unknown parameters

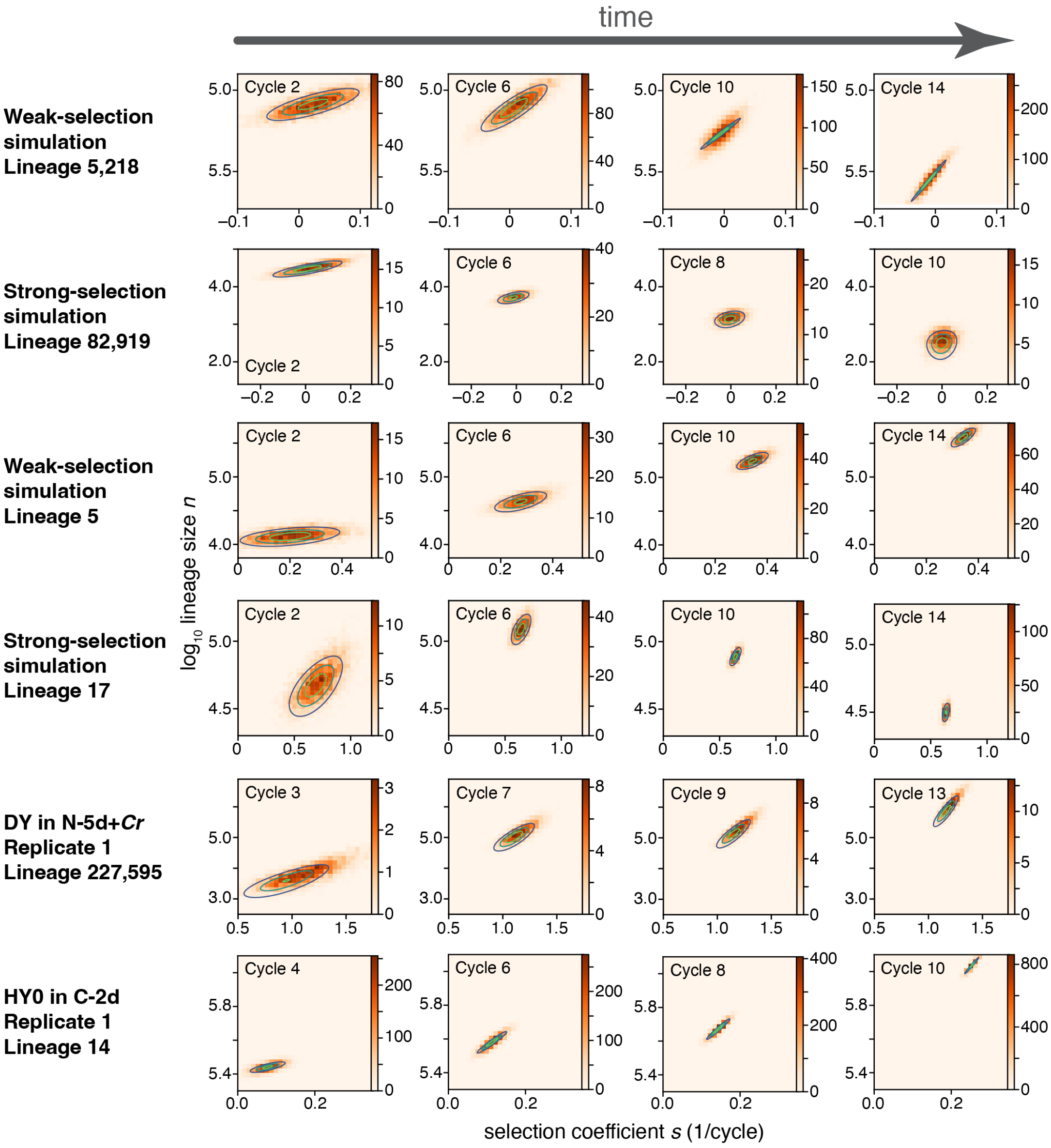

Figure B. Changes in the belief distribution as data is accumulated. Each row corresponds to a single lineage in a particular dataset, as indicated on the left. Note that the selection coefficient here is shown on the per cycle basis. Notations are as in Figure A.

$\bar{s}_k$ and ϵ_k, we can write down the log-likelihood function for $\bar{s}_k$ and ϵ_k as

$$L_k(\bar{s}, \epsilon) = \sum_{i \in \mathcal{A}_k} \log Z^{\text{sel}}(\boldsymbol{r}_{ik}; \bar{s}, \epsilon) + \sum_{i \in \mathcal{N}_k} \log Z^{\text{neut}}(\boldsymbol{r}_{ik}; \bar{s}, \epsilon), \tag{S63}$$

and estimate

$$(\bar{s}_k, \epsilon_k) = \arg\max_{\bar{s}, \epsilon} L_k(\bar{s}, \epsilon)$$

using standard optimization algorithms.

Classification of lineages for estimating mean fitness. We classify lineages into putatively neutral and putatively adapted to minimize estimation biases that may arise and be amplified over time due to the following positive feedback loop. Evolutionary dynamics in our model depend only on the difference between the fitness of a lineage and the mean fitness (see equations (S42),(S43)), but not on these two quantities individually. Thus, all selection coefficients and the mean fitness can be identified from data only up to a shared constant. We eliminate this ambiguity by assuming that the expected fitness of all lineages is zero at t_0 (see “Materials and Methods” in the main text). In other words, we estimate selection coefficients with respect to population’s initial mean fitness. While this condition should be sufficient to accurately estimate the mean fitness and lineage selection coefficients on average, the large uncertainty in our priors can lead to large uncertainties in the initial estimates of lineage fitness (Figure B) and mean fitness. These initial deviations can over time be “baked in” into the belief distributions because, as mentioned above, additional data can only correct any inaccuracies in the difference between lineage fitness and mean fitness but not in their individual values.

To make this reasoning more concrete, suppose that we do not classify lineages into putatively neutral and adapted and instead use lineage belief distributions to estimate mean fitness, i.e., use only Z^{sel} terms for all lineages in equation (S63). Imagine that to estimate mean fitness $\bar{s}_1$ at the first time interval (t_0, t_1), by chance we pick 3,000 lineages whose frequency declines (slightly) more than expected under neutrality. Then, we will (slightly) over-estimate $\bar{s}_1$. As a result, the projected distributions would under-estimate lineage sizes at t_1, thus generating an overly strong surprise once the read counts are observed. This would push us to update the belief distribution towards overly high s for many lineages, which would in turn cause an additional over-estimate of the mean fitness at the next time interval (t_1, t_2), etc.

To dampen this positive feedback loop at the mean-fitness estimation step, we assume that all lineages are neutral by default (for the purposes of mean-fitness estimation) unless we have a high degree of confidence that a given lineage is adapted. Specifically, we classify lineage i as putatively adapted for the purposes of estimating

$\bar{s}_k$ if

$$\mu_{ik-1} > 3\,\sigma_{ik-1}, \tag{S64}$$

where

$$\mu_{ik-1} = \int_{-\infty}^{\infty} ds \int_0^{\infty} s\, P^{\text{belief}}\left(n, s \,|\, \boldsymbol{r}_{ik-1}\right)\, dn,$$
$$\sigma^2_{ik-1} = \int_{-\infty}^{\infty} ds \int_0^{\infty} \left(s - \mu_{ik-1}\right)^2\, P^{\text{belief}}\left(n, s \,|\, \boldsymbol{r}_{ik-1}\right)\, dn$$

are the mean and variance of the marginal belief distribution for s for lineage i at time t_{k-1} estimated using equations (S48), (S49). The factor 3 in equation (S64) was chosen so that our confidence that the lineage is in fact adapted (i.e., has a positive s) exceeds 99%.

Neutral model of lineage evolution. To compute the marginal likelihood $Z^{\text{neut}}(\boldsymbol{r}_{ik}; \bar{s}_k, \epsilon_k)$ that r_{ik} reads are observed for lineage i at time point t_k if the lineage is neutral during the time interval (t_{k-1}, t_k), we employ a neutral model that is analogous to the model with selection described above in Sections 3.2.1 and 3.2.2.

As in the model with selection, we start with the belief distribution $P^{\text{belief}}\left(n, s \,|\, \boldsymbol{r}'\right)$, where $\boldsymbol{r}' = (r_0, \ldots, r_{k-1})$ is the observation vector up to and including the previous time point t_{k-1}. Since the neutral model is formulated only in terms of the lineage size n, we first need to obtain the marginal belief distribution $P^{\text{belief}}(n \,|\, \boldsymbol{r}')$. This distribution has no analytical expression. Therefore, to facilitate further calculations, we approximate it with a Gamma distribution with the scale parameter $\tilde{\theta}$ and shape parameter $\tilde{\varkappa}$ chosen to match the marginal mean and variance given by equations (S52), (S53) i.e., we set

$$P^{\text{belief}}(n \,|\, \boldsymbol{r}') = \gamma(n; \tilde{\varkappa}, \tilde{\theta}),$$
$$\text{with } \tilde{\varkappa} = \frac{\varkappa}{(1+\varkappa)e^{b^2} - \varkappa},$$
$$\text{and } \tilde{\theta} = \frac{a e^{\frac{b^2}{2}}}{\tilde{\varkappa}}.$$

We then obtain the prior distribution for the lineage size at the current time point t_k analogously to equation (S44),

$$\tilde{P}^{\text{prior}}(n) = \begin{cases} 1 - \tilde{p}_{\text{surv},L}\,, & \text{if } n = 0, \\ \tilde{p}_{\text{surv},L}\, \gamma\left(n; \tilde{\varkappa}_L, \tilde{\theta}_L\right), & \text{if } n > 0, \end{cases} \tag{S65}$$

where the L-cycle survival probability $\tilde{p}_{\text{surv},L}$ is given by equation (S45) with parameters $\tilde{\theta}_\ell = f_\theta\left(0, \tilde{\theta}_{\ell-1}, \tilde{\varkappa}_{\ell-1}\right)$, $\tilde{\varkappa}_\ell = f_\varkappa\left(\tilde{\theta}_{\ell-1}, \tilde{\varkappa}_{\ell-1}\right)$ for $\ell = 1, 2, \ldots L$ that are

obtained recursively using equations (S42) and (S43) with $\varkappa_0 \equiv \tilde{\varkappa}$, $\theta_0 \equiv \tilde{\theta}$. The marginal likelihood under the neutral model is then given by

$$Z^N(\boldsymbol{r}; \bar{s}_k, \epsilon_k) = \int_0^\infty \tilde{P}^{\text{prior}}(n \,|\, \boldsymbol{r}')\; P^{\text{meas}}(r_k \,|\, n)\, dn. \tag{S66}$$

Numerical estimation of the marginal likelihoods Z^{sel} and Z^{neut}. For every putatively adapted lineage, we estimate the marginal likelihood Z^{sel} given by equation (S47) by first obtaining a random sample (s_j, n_j), $j = 1, 2, \ldots, M$ with $M = 5,000$ from the projected distribution $P^{\text{prior}}(n, s; \bar{s})$ for that lineage, which is given by equation (S44), using a standard random-number generator, keeping only those samples with $n_j > 0$, i.e., conditional on lineage survival. We then calculate

$$\hat{Z}^{\text{sel}}(\boldsymbol{r}; \bar{s}, \epsilon) = \frac{1}{M} \sum_{j=1}^{M} P^{\text{meas}}(r_k \,|\, n_j; \epsilon). \tag{S67}$$

For every putatively neutral lineage, we estimate the marginal likelihood Z^{neut} given by equation (S66) analogously, by first obtaining a random sample n_j, $j = 1, 2, \ldots, M$ from the projected distribution $\tilde{P}^{\text{prior}}(n; \bar{s})$ given by equation (S65), keeping only those samples with $n_j > 0$. We then estimate $\hat{Z}^{\text{neut}}$ as per equation (S67).

3.4 Identification of adapted lineages and estimation of their selection coefficients

As a result of applying BASIL, we obtain a time-varying belief distribution $P_{ik}^{\text{belief}}(n, s) \equiv P_{ik}^{\text{belief}}(n, s | \boldsymbol{r}_{ik})$ for each lineage i at each sampling time point t_k, $k = 1, 2, \ldots$. Our primary interest is in the marginal belief distribution for the selection coefficient s, $P_{ik}^{\text{belief}}(s) \equiv P^{\text{belief}}(s | \boldsymbol{r}_{ik})$ whose mean and variance are μ_{ik} and σ_{ik}^2, estimated using equations (S48), (S49). At early time points, we expect these belief distributions to have high variance because they are insufficiently constrained by the data. As data accumulates, the uncertainty in s should decline, particularly for lineages that actually acquired a single adaptive mutation (Figure B). However, if we wait long enough, secondary adaptive mutations might appear, which could again increase the uncertainty in our belief of s. Thus, for each lineage i, we find the time point $t_{k'(i)}$ with minimal variance, $k'(i) = \arg\min_k \hat{\sigma}_{ik}^2$. We call lineage i adapted if $\hat{\mu}_{ik'(i)} > \beta\, \hat{\sigma}_{ik'(i)}$ where β is the "confidence factor", a hyper-parameter that controls the precision and recall of our inference. As described in the main text, we empirically determine that the confidence factor $\beta = 3.3$ maximizes the F1-score (the harmonic mean of precision and recall) in our simulated data, and use this value for all our analyses. For each lineage i called as adapted, we estimate its selection coefficient as $\hat{s}_i = \hat{\mu}_{ik'(i)}$, and we can calculate the credible interval for it using the standard deviation $\hat{\sigma}_i = \hat{\sigma}_{ik'(i)}$.

Algorithm A BASIL

procedure CONSTRUCT SELECTION MODEL ▷ Sec. 3.2

procedure CONSTRUCT NEUTRAL MODEL ▷ Sec. 3.3

procedure ESTIMATE BELIEF DISTRIBUTIONS

  for all lineage i **do**

    procedure INITIALIZE BELIEF DISTRIBUTION ▷ Materials and Methods in the main text

      $P^{\text{belief}}(n, s \,|\, r_{i0}) = N(s; \mu, \sigma)\, \gamma\, (n; \varkappa(r_{i0}), \theta(r_{i0}))$

      with $\mu = 0$, $\sigma = 0.1$, $\varkappa = r_{i0} + 1$, $\theta = (R_0 + N_L)^{-1}$

  for time point $k = 1, 2, \ldots, k_{\text{final}}$ **do**

    procedure ESTIMATE MEAN FITNESS FOR (t_{k-1}, t_k) ▷ Sec. 3.3

      $\bar{s}_k = \arg\max_{\bar{s}} L_k(\bar{s})$ ▷ Eq. (S63)

    for all lineage i **do**

      procedure OBTAIN PRIOR DISTRIBUTION FOR t_k ▷ Sec. 3.2.1

        $P^{\text{belief}}(n, s \,|\, \mathbf{r}_{ik-1}) \to P^{\text{prior}}(n, s \,|\, \mathbf{r}_{ik-1})$ ▷ Eq. (S44)

      procedure UPDATE BELIEF DISTRIBUTION AT t_k ▷ Sec. 3.2.2

        $P^{\text{prior}}(n, s \,|\, \mathbf{r}_{ik-1}) \to P^{\text{belief}}(n, s \,|\, \mathbf{r}_{ik})$ ▷ Eq. (S46)

procedure IDENTIFY ADAPTED LINEAGES ▷ Sec. 3.4

procedure ESTIMATE SELECTION COEFFICIENTS ▷ Sec. 3.4

3.5 Implementation

Figure 3 in the main text and Algorithm A show the overall BASIL workflow. We implement BASIL in a Python-based software package. For MCMC sampling, this package uses the C library *stan* and the Python package *pystan 2* as an interface between Python and *stan*. MCMC sampling is implemented using the No-U-Turn Sampler (NUTS) sampler, which efficiently generates proposals based on the posterior distribution [4]. For each belief distribution, we obtain $M = 3500$ samples, with a burn-in of 1,000 steps. For the maximum likelihood estimation of mean fitness, we use the Python package *Noisyopt* for the optimization of noisy functions. Noise arise from the fact that we estimate $\hat{Z}^{\text{sel}}$ and $\hat{Z}^{\text{neut}}$ for every lineage using a Monte Carlo method as described in Section 3.3. The *Noisyopt* package repeatedly evaluates the stochastic target function, then averages over the stochasticity to ensure convergence.

The BASIL analysis of a single BLT experiment with 5×10^5 lineages sampled at 10 time points took us about 50 hours to complete on AMD Ryzen 5 7600X 6-Core Processor, and we found that the runtime increases linearly in the number of lineages and sampling time points. However, since MCMC samples for different lineages are independent at each time point, our algorithm is easily parallelizable, and a multiprocessing capability has been implemented in BASIL. The number of processors can be set manually depending on the user's environment. In our analyses, we found it convenient to use 12 to 32 processor cores.

BASIL code, example data and installation instructions are available at

https://github.com/HuanyuKuo/BASIL-public

When barcode read count data are provided, the algorithm automatically detects the total number of barcodes and the cycle duration between sampling times. Other parameters can be adjusted in myConstant.py. These include:

- Biological settings: dilution factor, carrying capacity, and the number of randomly chosen reference lineages for mean fitness estimation.
- System settings: number of processors (CPU cores) used for parallel computation.

For adapted lineage calling, we use a confidence factor of $\beta = 3.3$ as the default setting in this work. However, users may adjust β to explore the sensitivity of lineage calls (see Discussion in the main text and Figure S10). Since β only affects the final lineage-calling step, changing its value does not require re-running the time-consuming MCMC estimation.

After completing a run, BASIL generates the following output files:

- Bayesian_global_parameters_XXX: inferred mean fitness trajectory and inferred ϵ trajectory.
- BASIL_Selection_Coefficient_for_called_Adapted_XXX: list of adapted lineages, including barcode indices, estimated selection coefficients (s) with mean and standard deviation, and the calling time.
- posterior_XXX_SModel_S_T1: the parametric belief distributions of all lineages at a particular sampling time point.
- glob_XXX_T1: information of maximizing the likelihood of the mean fitness and ϵ at a particular sampling time point.

We recommend that users first run BASIL on the provided example BLT dataset. This test run helps verify that the installation is working properly and familiarizes users with the workflow before applying the algorithm to their own data.

4 Application of BASIL to simulated data

4.1 Belief distribution and lineage identification

Figure B shows how the belief distribution $P_{ik}^{\text{belief}}(n, s)$ changes over time as information about the lineage frequency is accrued. Next, as discussed in the main text, we used simulated data to determine the optimal value of the confidence factor β, setting $\beta = 3.3$. The confidence factor has a simple interpretation. We use a linear classifier to classify lineages into adapted and neutral. Specifically, if we plot the inferred selection coefficient of each lineage $\hat{s}_i$ and the estimated standard deviation $\hat{\sigma}_i$ of the credible interval around it, then $1/\beta$ is the slope of the line that separates adapted from non-adapted lineages (see Figures S3). We find that lineages form two large clusters (with some finer clustering structure visible in Figures S3B) that contain overwhelmingly adapted or non-adapted lineages, respectively. The classification line $\hat{s}_i = \beta\, \hat{\sigma}_i$ with $\beta = 3.3$ separates these two clusters very well (see Table 1 in the main text).

4.2 Performance on simulated data

To analyze the performance of BASIL on lineage identification, we calculate the following standard statistics:

- The number of true positives (TP), i.e., adapted lineages identified as such;
- The number of false positives (FP), i.e., neutral lineages incorrectly identified as adapted;

- The number of true negatives (TN), i.e., neutral lineages identified as such;
- The number of false negatives (FN), i.e., adapted lineages incorrectly identified as neutral;
- Precision, i.e., the fraction of all positives that are true, TP/(TP+FP);
- Recall, or the true positive rate, i.e., the fraction of all adapted lineages that are identified as such, TP/(TP+FN);
- The F1-score, which is the harmonic mean of precision and recall.

All numbers and statistics are reported in Table 1 in the main text. In particular, in the weak selection region, we obtain 31 false positives (precision 98.8%) and 430 false negatives (recall 85.7%), and in the strong selection region, we obtain 7 false positives (precision 99.7%) and 519 false negatives (recall 82.7%). Figure S3 reveals that all false positives, i.e., neutral lineages that are incorrectly identified as adapted, are very close to the classification line, suggesting that in principle their rate could be further reduced by increasing β (albeit at the expense of increasing the rate of false negatives). In contrast, false negatives, i.e., adapted lineages that are incorrectly classified as neutral, are distributed broadly within the non-adapted cluster, indicating that no linear classifier that is based solely on $\hat{s}_i$ and $\hat{\sigma}_i$ can achieve a substantially higher recall. This observation suggests that false negative lineages may be statistically indistinguishable from true neutral ones.

To investigate this conjecture, we first plotted the trajectories of all lineages stratified by their predicted and actual class labels. Figures S4 and S5 confirm that the trajectories of false negative lineages are visually indistinguishable from those of truly neutral lineages. One possible explanation for why some adapted lineages behave is if they are neutral is that these lineages have such small population sizes that their dynamics are governed largely by genetic drift, that is, they fail to "establish" [5]. An adapted lineage establishes approximately when its size n_i exceeds the inverse of its selection coefficient $1/s_i$, or equivalently, $n_i s_i > 1$ [5]. Thus, lineages with smaller selection coefficients have a smaller chance of successfully establishing. Consistent with this prediction, we find that false negative lineages have significantly smaller selection coefficients than true positive lineages, with $P < 0.001$ in both cases (Welch's t-test; see Figures S6). Next, for each lineage i, we estimated the maximum size it has achieved during the course of the simulation as $\tilde{n}_i = \max_k r_{ik} N_b / R_k$ and plotted the lineage's true selection coefficient s_i against $\tilde{n}_i$. Figure S6 show that for the majority of false negative lineages, we have $\tilde{n}_i s_i < 1$, whereas for the vast majority of true positive lineages, $\tilde{n}_i s_i > 1$, as expected. These observations support our hypothesis that some adapted lineages fail to establish. As a result, their dynamics are highly stochastic and essentially indistinguishable from neutral.

Appendix A Expectation of the noise variance estimator and bias correction

Since the estimate (S4) we calculate its expectation to determine whether it is biased.

$$\mathbb{E}\left(\hat{\sigma}_{pi}\right) = \frac{1}{|X_p|}\sum_{b\in X_p}\left(\mathbb{E}\left(r_{bi}^2\right) - 2\mathbb{E}\left(r_{bi}^2\,\bar{r}_{bi}^2\right) + \mathbb{E}\left(\bar{r}_{bi}^2\right)\right). \tag{S68}$$

To calculate the expectations in the sum of equation (S68), note that

$$\mathbb{E}\left(r_{ib}\, r_{jb}\right) = p_b^2 R_i R_j + \delta_{ij}\,\sigma_{pi}^2.$$

Now, we have

$$\mathbb{E}\left(r_{bi}^2\right) = \left(p_b R_i\right)^2 + \sigma_{pi}^2, \tag{S69}$$

$$\mathbb{E}\left(r_{bi}^2\,\bar{r}_{bi}^2\right) = \frac{R_i}{n_{\text{rep}}}\sum_{j=1}^{n_{\text{rep}}}\frac{\mathbb{E}\left(r_{bi}\, r_{bj}\right)}{R_j} = \left(p_b R_i\right)^2 + \frac{\sigma_{pi}^2}{n_{\text{rep}}}, \tag{S70}$$

$$\mathbb{E}\left(\bar{r}_{bi}^2\right) = \left(\frac{R_i}{n_{\text{rep}}}\right)^2\sum_{j,k=1}^{n_{\text{rep}}}\frac{\mathbb{E}\left(r_{bj}\, r_{bk}\right)}{R_j R_k} = \left(p_b R_i\right)^2 + \left(\frac{R_i}{n_{\text{rep}}}\right)^2\sum_{j=1}^{n_{\text{rep}}}\frac{\sigma_{pj}^2}{R_j^2}. \tag{S71}$$

Substituting equations (S69)–(S71) into equation (S68), we obtain

$$\mathbb{E}\left(\hat{\sigma}_{pi}\right) = \sigma_{pi}^2\left(1 - \frac{1}{n_{\text{rep}}}\right)^2 + \left(\frac{R_i}{n_{\text{rep}}}\right)^2\sum_{j\neq i}\frac{\sigma_{pj}^2}{R_j^2}. \tag{S72}$$

Thus, the estimate (S4) is a biased estimate of σ_{pi}^2. Note that if all replicates have the same coverage, $R_i = R$, and $\sigma_{pi}^2 = \sigma_p^2$, equation (S72) simplifies to

$$\mathbb{E}\left(\hat{\sigma}_{pi}\right) = \sigma_p^2\left[\left(1 - \frac{1}{n_{\text{rep}}}\right)^2 + \frac{n_{\text{rep}} - 1}{n_{\text{rep}}^2}\right] = \sigma_p^2\left(1 - \frac{1}{n_{\text{rep}}}\right),$$

and correcting the bias is easy. To correct the bias in the general case, we rewrite equation (S72) in the matrix form as

$$\mathbb{E}\left(\boldsymbol{V}_p\right) = A\,\boldsymbol{\sigma}_p^2,$$

where $\boldsymbol{V}_p = \left(\hat{\sigma}_{p1}, \hat{\sigma}_{p2}, \ldots, \hat{\sigma}_{pn_{\text{rep}}}\right)^T$ and $\boldsymbol{\sigma}_p^2 = \left(\sigma_{p1}^2, \sigma_{p2}^2, \ldots, \sigma_{pn_{\text{rep}}}^2\right)^T$ and

$$A = \begin{pmatrix} \left(1 - \frac{1}{n_{\text{rep}}}\right)^2 & \frac{1}{n_{\text{rep}}^2}\frac{R_1^2}{R_2^2} & \cdots & \frac{1}{n_{\text{rep}}^2}\frac{R_1^2}{R_{n_{\text{rep}}}^2} \\ \frac{1}{n_{\text{rep}}^2}\frac{R_2^2}{R_1^2} & \left(1 - \frac{1}{n_{\text{rep}}}\right)^2 & \cdots & \frac{1}{n_{\text{rep}}^2}\frac{R_2^2}{R_{n_{\text{rep}}}^2} \\ \vdots & \vdots & \ddots & \vdots \\ \frac{1}{n_{\text{rep}}^2}\frac{R_{n_{\text{rep}}}^2}{R_1^2} & \frac{1}{n_{\text{rep}}^2}\frac{R_{n_{\text{rep}}}^2}{R_2^2} & \cdots & \left(1 - \frac{1}{n_{\text{rep}}}\right)^2 \end{pmatrix},$$

and obtain the bias-corrected estimate

$$\boldsymbol{V}'_p = A^{-1}\boldsymbol{V}_p. \tag{S73}$$

Appendix B Expected frequency given the number of reads

Here, we derive the expressions (S22)–(S28) for the conditional expected frequency of a lineage $\mathbb{E}\left(x|r\right)$, given the number of reads r.

Example 1. Poisson measurement noise and a uniform frequency distribution. We first consider the case when the measurement process is modeled by the Poisson distribution (S21) and the frequency distribution is uniform. Then, the conditional distribution for the frequency x is given by

$$P(x|r)\,dx = \frac{P^{\text{meas}}(r|x)\,P^{\text{prior}}(x)}{P(r)}\,dx = C\,x^r\,e^{-xR}\,dx, \quad \text{with } x \in [0,1], \tag{S74}$$

where C is the normalization constant. As long as $r/R \ll 1$, this distribution is well approximated by the gamma distribution with the shape parameter $k = r+1$ and scale parameter $\theta = 1/R$. Thus, $C = R^{r+1}/r!$ whose mean is $k\theta = (r+1)/R$ confirming (S22).

Example 2. Poisson measurement noise and an exponential frequency distribution. If the frequency distribution is exponential with mean $1/N_L$ (see equation (S23)), then we have

$$P(x|r)\,dx = C\,x^r\,e^{-x(R+N_L)}\,dx, \tag{S75}$$

which is again is very well approximated by the gamma distribution with the shape parameter $k = r+1$ and scale parameter $\theta = 1/(R+N_L)$, as long as $r \ll R$. Expression (S24) follows immediately.

Example 3. Measurement noise with an increasing variance to mean ratio and a Gamma frequency distribution. We next consider the case when the frequency distribution is Gamma with shape parameter α and scale parameter $1/\tilde{N}$ (see equation (S26)) and the read count is a negative binomial random variable with mean xR and the variance to the mean ratio $1+\epsilon xR$ (see (S25)). When $xR \ll 1/\epsilon$, the negative binomial distribution (S25) converges to the Poisson distribution with mean xR, which implies that the conditional distribution for x is a Gamma

distribution with shape parameter $r+\alpha$ and scale parameter $(R+\tilde{N})^{-1}$. More generally, the conditional distribution for x given r is given by

$$P(x|r)dx = \frac{P^{\text{meas}}(r|x)\,P^{\text{prior}}(x)}{P(r)}dx = C\,x^{r+\alpha-1}(1+\epsilon Rx)^{-(1/\epsilon+r)}\,e^{-\tilde{N}x}\,dx. \tag{S76}$$

It is possible to use Laplace's method to obtain approximate expressions for the normalization constant C and the moments of this distribution. Specifically, whenever $\epsilon \ll 1$, we obtain

$$C = \frac{\tilde{N}+R}{\sqrt{8\pi(r+\alpha-1)}}\,\frac{(1+\epsilon R\bar{x}_0)^{\frac{1}{\epsilon}+r}}{\bar{x}_0^{\alpha+r+1}}\,e^{\tilde{N}\bar{x}_0}$$
$$\mathbb{E}\left(x^i|r\right) = (\bar{x}_i)^i\left(\frac{r+\alpha+i-1}{r+\alpha-1}\right)^{r+\alpha-\frac{1}{2}}\left(\frac{1+\epsilon R\bar{x}_0}{1+\epsilon R\bar{x}_i}\right)^{\frac{1}{\epsilon}+r}e^{-\frac{\tilde{N}i}{\tilde{N}+R}},$$

for $i, r = 1, 2, \ldots$, where $\bar{x}_i = \frac{r+\alpha+i-1}{\tilde{N}+R}$.

Example 4. Measurement noise with a constant variance to mean ratio and an exponential frequency distribution. Finally, we consider the case when the measurement process is over-dispersed but with a constant variance to the mean ratio 2κ (see equation (S25)) and the frequency distribution is exponential (see equation (S23)). To facilitate analytical tractability, instead of tracking frequency x of the lineage in the population, we will track its lineage size n. We assume that n take values $1, 2, \ldots$ and that its prior distribution is geometric with parameter q. The expected lineage size is N/N_L, which implies that $q = N_L/N$. Thus, for the conditional distribution for n, given r is

$$P(n|r) = \frac{P^{\text{meas}}(r|n)\,P^{\text{prior}}(n)}{P(r)} = \frac{1}{P(r)}\,\frac{\Gamma(r+k)}{\Gamma(r+1)\Gamma(k)}(1-p)^r p^k\,(1-q)^{n-1}q, \tag{S77}$$

where $p = 1/2\kappa$, $k = \alpha n$, $q = N_L/N$ and $\alpha = R/N(2\kappa-1)^{-1}$. Expression (S77) suggests that the quantity $\tilde{k} = \alpha n - 1$ is a negative binomial random variable with parameters

$$\tilde{r} = r+1, \tag{S78}$$
$$\tilde{p} = 1 - p\,(1-q)^{1/\alpha},$$

that is,

$$P(\tilde{k}|r) = C\frac{\Gamma\left(\tilde{k}+\tilde{r}\right)}{\Gamma\left(\tilde{k}+1\right)}\,(1-\tilde{p})^{\tilde{k}}, \tag{S79}$$

where $C = \tilde{p}^{\tilde{r}}/\Gamma(\tilde{r})$ is the normalization constant. Thus, we have

$$\mathbb{E}\left(\tilde{k}\middle|r\right) = \frac{\tilde{r}(1-\tilde{p})}{\tilde{p}} = \frac{p\,(r+1)\,(1-q)^{1/\alpha}}{1-p\,(1-q)^{1/\alpha}}.$$

Since $\tilde{k} = x\frac{R}{2\kappa-1} - 1$, we have

$$\mathbb{E}\left(x|r\right) = \frac{2\kappa-1}{R}\frac{2\kappa + r\,(1-q)^{1/\alpha}}{2\kappa - (1-q)^{1/\alpha}} \approx \frac{2\kappa + r}{R},$$

where the last approximation holds because $q = N_L/N \sim 0.01 \ll 1$.

It will also be useful to calculate the expected number of cells in a lineage whose measured number of reads is r,

$$\mathbb{E}\left(n|r\right) = \frac{\mathbb{E}\left(\tilde{k}|r\right)+1}{\alpha} \approx \frac{N}{R}\left(2\kappa + r\right), \tag{S80}$$

and its variance

$$\begin{aligned}\mathrm{Var}\left(n|r\right) &= \frac{1}{\alpha^2}\,\mathrm{Var}\left(\tilde{k}|r\right) = \frac{1}{\alpha^2}\,\frac{\tilde{r}(1-\tilde{p})}{\tilde{p}^2} \\ &= \frac{1}{\alpha^2}\,\frac{(r+1)p\,(1-q)^{1/\alpha}}{(1-p\,(1-q)^{1/\alpha})^2} \approx \frac{N^2}{R^2}\,2\kappa\,(r+1)\,. \end{aligned} \tag{S81}$$

Appendix C Expectation of the logarithm of a gamma-distributed random variable

Consider a random variable X that is Gamma-distributed with scale parameter θ and shape parameter $\varkappa$, such that its probability density is

$$p(x) = \frac{1}{\Gamma(\varkappa)\,\theta^{\varkappa}}\,x^{\varkappa-1}\,e^{-\frac{x}{\theta}}.$$

Therefore, we have

$$\int_0^\infty x^{\varkappa-1}\,e^{-\frac{x}{\theta}}\,dx = \Gamma(\varkappa)\,\theta^{\varkappa}. \tag{S82}$$

Differentiating both sides of equation (S82) with respect to $\varkappa$, we obtain

$$\int_0^\infty \ln x\, x^{\varkappa-1}\,e^{-\frac{x}{\theta}}\,dx = \Gamma(\varkappa)\,\theta^{\varkappa}\left(\frac{\Gamma'(\varkappa)}{\Gamma(\varkappa)} + \ln\theta\right),$$

which can be rewritten as

$$\mathbb{E}\left(\ln X\right) = \psi(\varkappa) + \ln\theta,$$

where $\psi(\varkappa) = \frac{\Gamma'(\varkappa)}{\Gamma(\varkappa)}$ is the digamma function.

References

[1] Johnson MS, Venkataram S, Kryazhimskiy S. Best practices in designing, sequencing, and identifying random DNA barcodes. J Mol Evol. 2023;91(3):263–280.

[2] Levy SF, Blundell JR, Venkataram S, Petrov DA, Fisher DS, Sherlock G. Quantitative evolutionary dynamics using high-resolution lineage tracking. Nature. 2015;519(7542):181–186.

[3] Li F, Mahadevan A, Sherlock G. An improved algorithm for inferring mutational parameters from bar-seq evolution experiments. BMC Genomics. 2023;24(1):246.

[4] Hoffman MD, Gelman A, et al. The No-U-Turn sampler: adaptively setting path lengths in Hamiltonian Monte Carlo. J Mach Learn Res. 2014;15(1):1593–1623.

[5] Desai MM, Fisher DS. Beneficial mutation-selection balance and the effect of linkage on positive selection. Genetics. 2007;176(3):1759–1798.
