# Supplementary material for: Putting BASIL in a BLT: A Bayesian filtering method for estimating the fitness effects of nascent adaptive mutations
Source: PLoS Comput Biol. 2026 Feb 27;22(2):e1013946. doi: 10.1371/journal.pcbi.1013946 (PMC12974954; doi:10.1371/journal.pcbi.1013946)
Supplement: S1 Fig — (PDF) [file pcbi.1013946.s002.pdf]

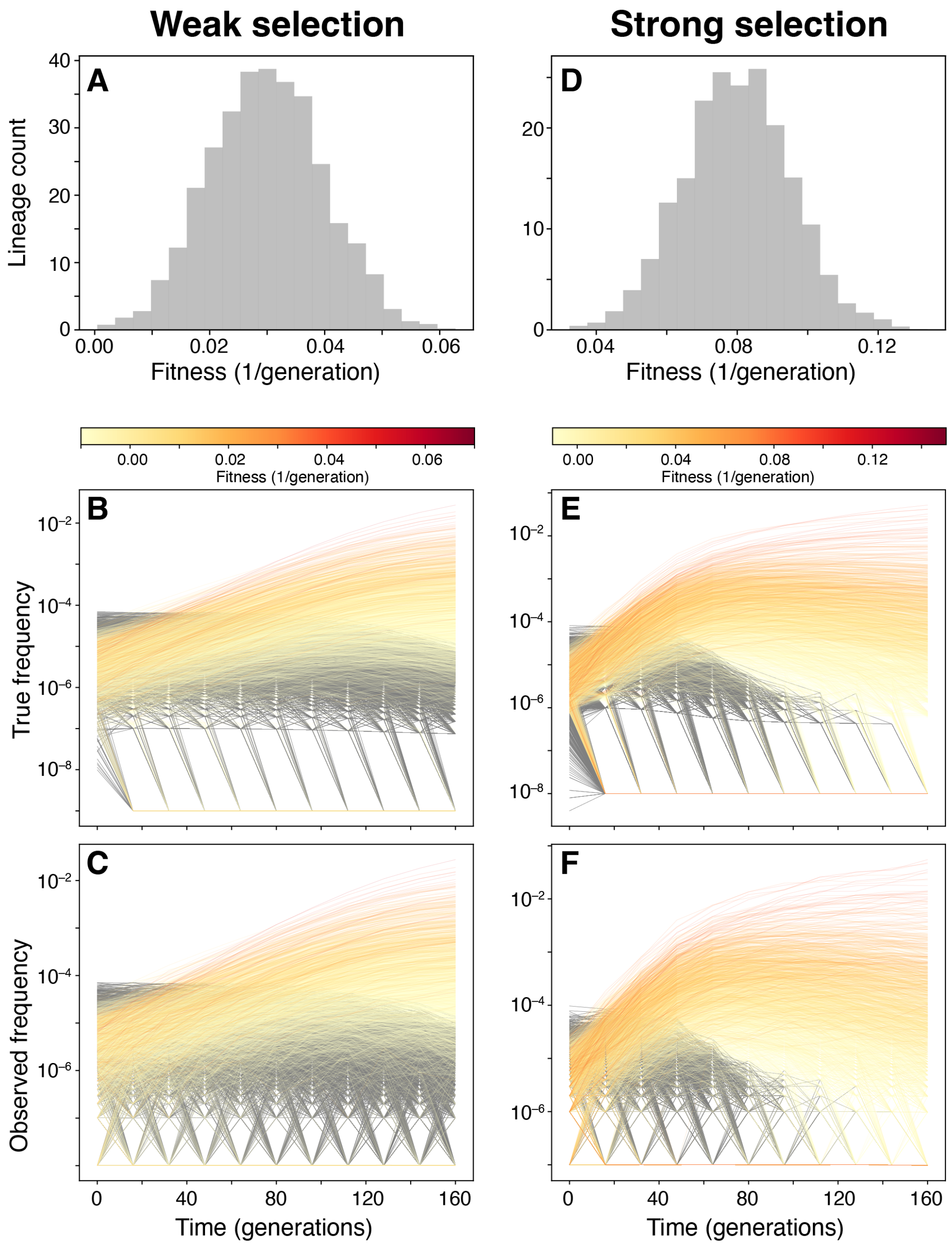

Figure S1. Simulated data. A–C. Weak selection regime. **D–F.** Strong selection regime. Distributions of lineage fitness (A,D). True lineage frequency trajectories (B,E). Observed barcode frequency trajectories (C,F). Adapted lineages are colored by their fitness, neutral lineages are gray.
