# Supplementary material for: Putting BASIL in a BLT: A Bayesian filtering method for estimating the fitness effects of nascent adaptive mutations
Source: PLoS Comput Biol. 2026 Feb 27;22(2):e1013946. doi: 10.1371/journal.pcbi.1013946 (PMC12974954; doi:10.1371/journal.pcbi.1013946)
Supplement: S2 Fig — (PDF) [file pcbi.1013946.s003.pdf]

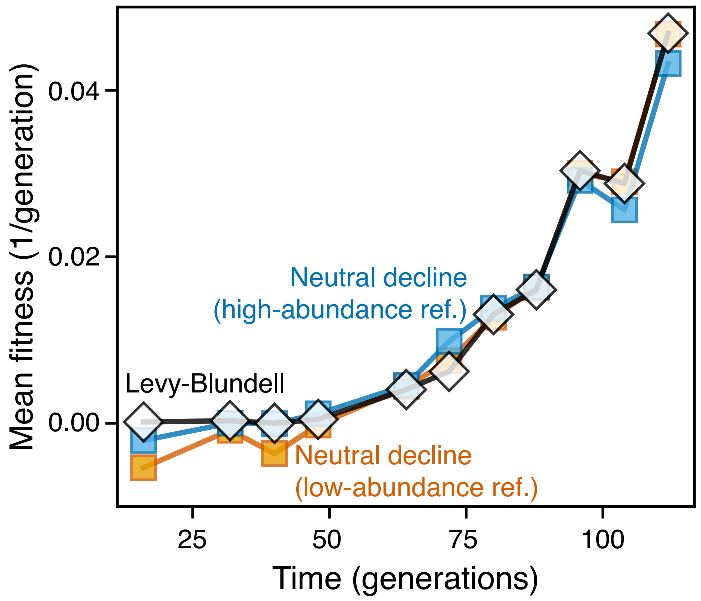

Figure S2. Correspondence between the full Levy-Blundell inference method and the simplified neutral decline method. Mean-fitness trajectories in Replicate 1 in the Levy 2015 dataset as reported in Ref. [3] (black line and white diamonds), and inferred by our the neutral decline method using either high-abundance lineages (blue line and squares) or low-abundance lineages (orange line and squares) as reference.
