# Supplementary material for: Putting BASIL in a BLT: A Bayesian filtering method for estimating the fitness effects of nascent adaptive mutations
Source: PLoS Comput Biol. 2026 Feb 27;22(2):e1013946. doi: 10.1371/journal.pcbi.1013946 (PMC12974954; doi:10.1371/journal.pcbi.1013946)
Supplement: S3 Fig — (PDF) [file pcbi.1013946.s004.pdf]

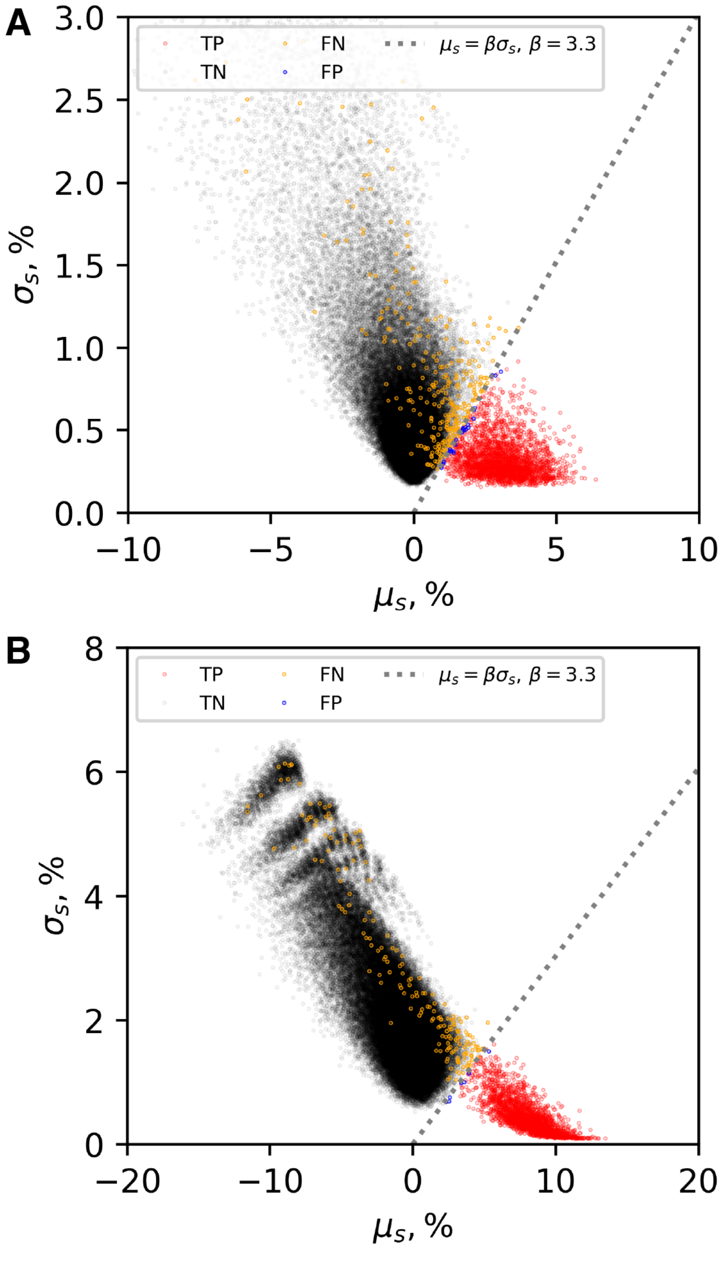

Figure S3. Classification of lineages in simulated data. A. Mean (x axis) versus the standard deviation (y axis) of the estimated marginal belief distribution for the lineage selection coefficient for simulated data in the weak selection regime. **B.** Same for the strong-selection regime. Each point represents a lineage. Colors represent different lineage classes: red = true positives, black = true negatives, orange = false negatives, blue = false positives. Dashed line is the classification line with $\beta = 3.3$.
