# Supplementary material for: Putting BASIL in a BLT: A Bayesian filtering method for estimating the fitness effects of nascent adaptive mutations
Source: PLoS Comput Biol. 2026 Feb 27;22(2):e1013946. doi: 10.1371/journal.pcbi.1013946 (PMC12974954; doi:10.1371/journal.pcbi.1013946)
Supplement: S4 Fig — (PDF) [file pcbi.1013946.s005.pdf]

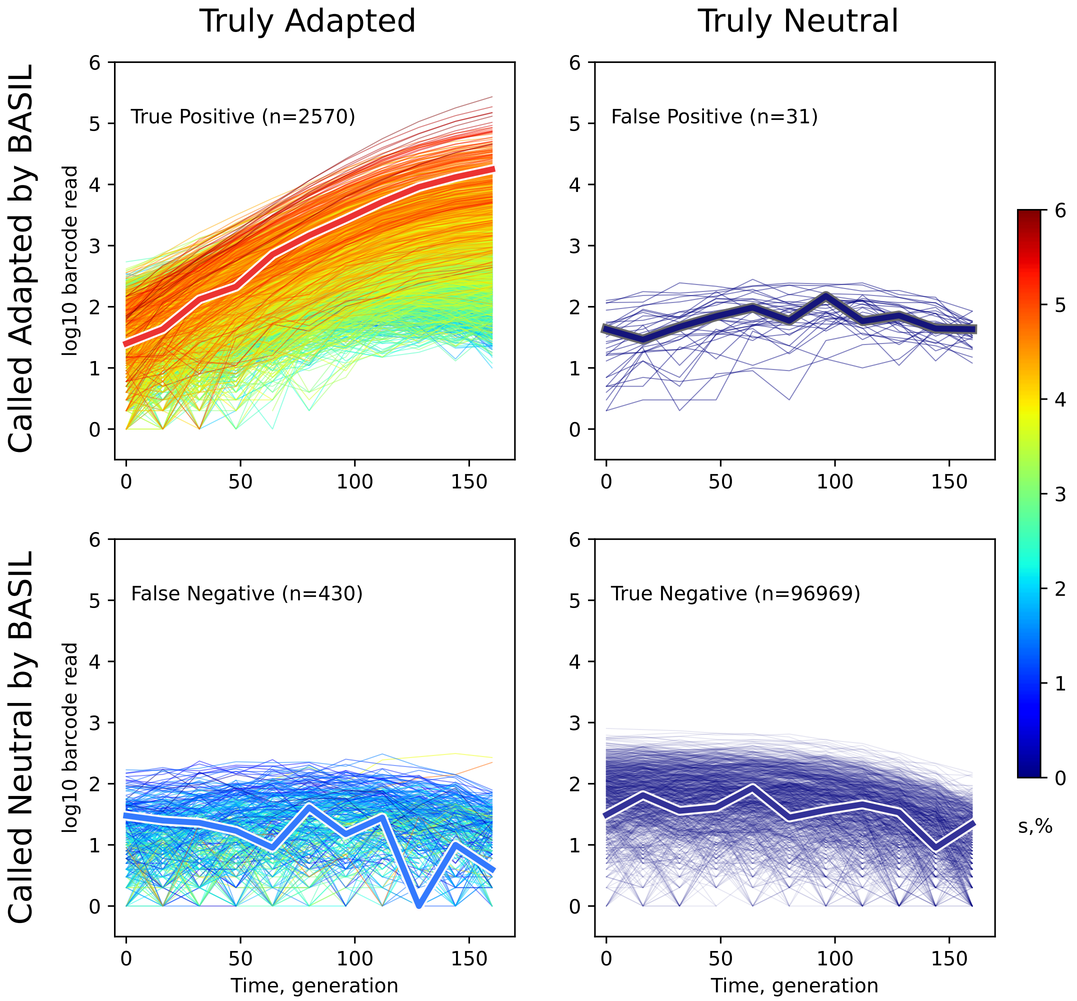

Figure S4. Lineage trajectories in the weak-selection simulation stratified by class. Data is the same as in Figure S1B. Only 970 random true negative lineages are shown to improve visualization. Trajectories are colored by lineage fitness.
