# Supplementary material for: Putting BASIL in a BLT: A Bayesian filtering method for estimating the fitness effects of nascent adaptive mutations
Source: PLoS Comput Biol. 2026 Feb 27;22(2):e1013946. doi: 10.1371/journal.pcbi.1013946 (PMC12974954; doi:10.1371/journal.pcbi.1013946)
Supplement: S6 Fig — (PDF) [file pcbi.1013946.s007.pdf]

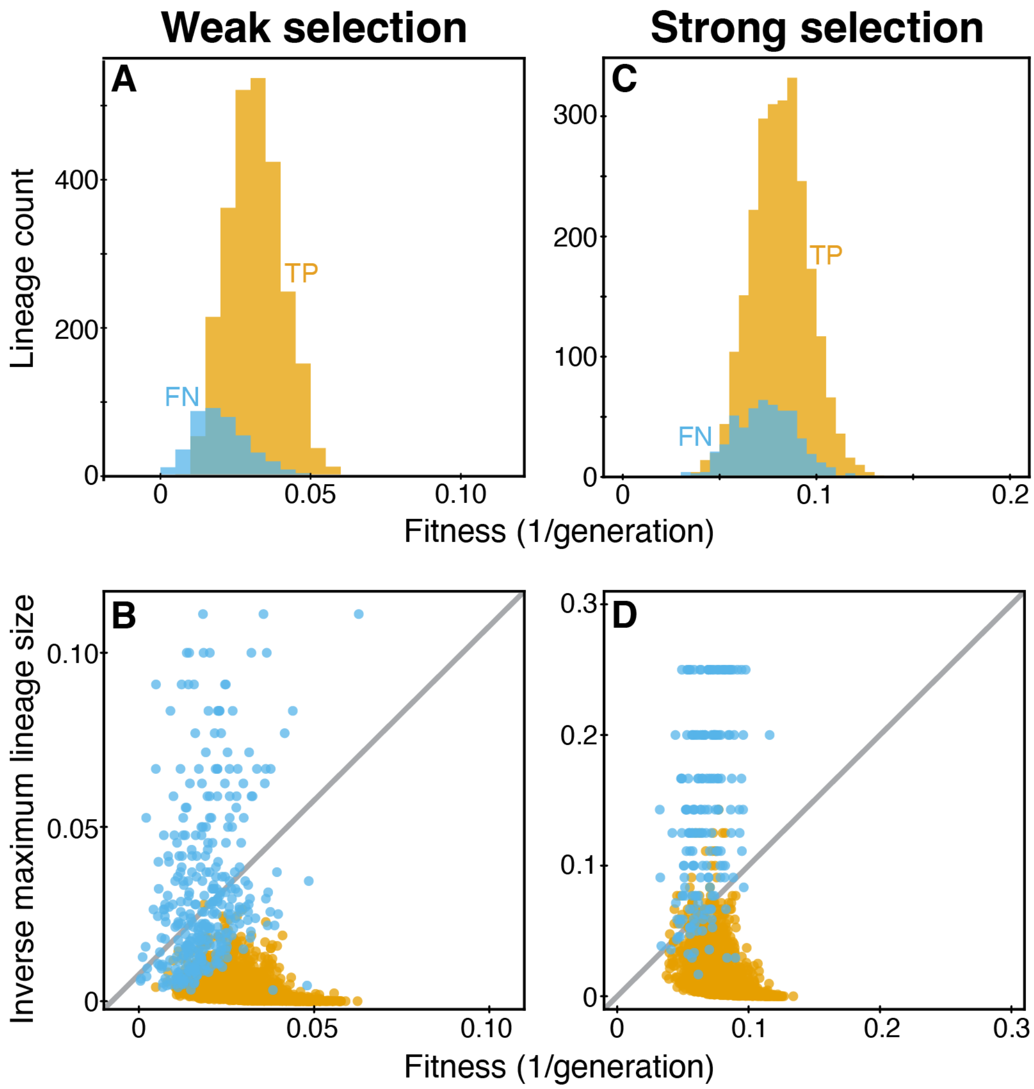

Figure S6. True positive and false negative lineages have distinct features in simulated data. A. Distribution of selection coefficients of true positives (yellow) and false negative (blue) lineages in the weak-selection simulation. **B.** The relationship between lineage fitness s_i and its maximum size $\tilde{n}_i$ in the simulation (see Section 4.2 in Text S1 for details). Lineages above the diagonal (gray line) are those with $\tilde{n}_i s_i < 1$, and lineages below the diagonal are those with $\tilde{n}_i s_i > 1$. **C.** Same as panel A but for the strong-selection simulation. **D.** Same as panel B but for the strong-selection simulation.
