# Supplementary material for: Putting BASIL in a BLT: A Bayesian filtering method for estimating the fitness effects of nascent adaptive mutations
Source: PLoS Comput Biol. 2026 Feb 27;22(2):e1013946. doi: 10.1371/journal.pcbi.1013946 (PMC12974954; doi:10.1371/journal.pcbi.1013946)
Supplement: S7 Fig — (PDF) [file pcbi.1013946.s008.pdf]

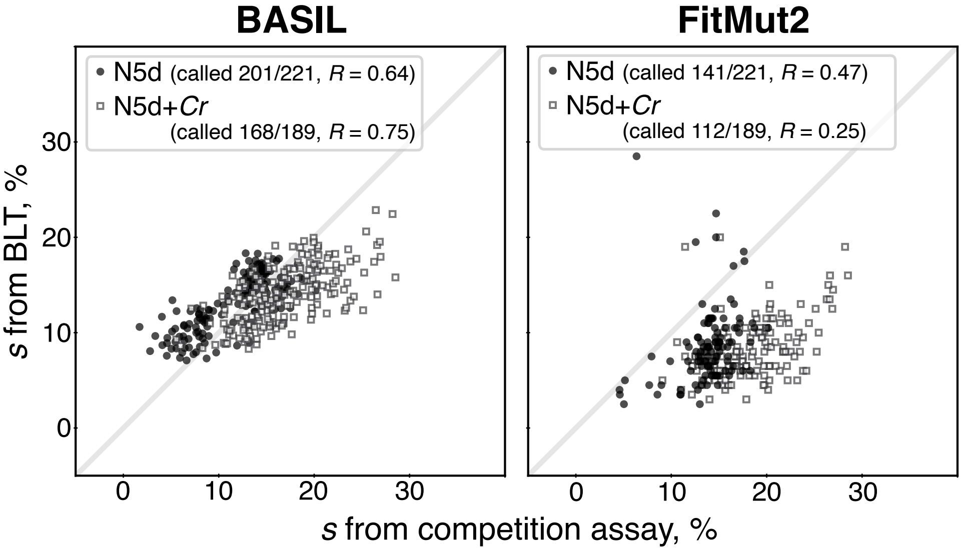

Figure S7. Comparison of BASIL and FitMut2 performance on Venkataram 2023 data. Lineage fitness inferred from BLT experiments by either BASIL or FitMut2 is plotted against the fitness of respective isolated clones measured in competition assays by Venkataram et al [33]. Filled circles represent lineages/clones measured in the N5d condition, which Venkataram et al refer to as "Alone". Empty squares represent lineages/clones measured in the N5d+*Cr* condition, which Venkataram et al refer to as "Community". The number of lineages called as adapted in the respective BLT experiment as well as Pearson correlation coefficient R are shown in parenthesis.
