# Supplementary material for: Putting BASIL in a BLT: A Bayesian filtering method for estimating the fitness effects of nascent adaptive mutations
Source: PLoS Comput Biol. 2026 Feb 27;22(2):e1013946. doi: 10.1371/journal.pcbi.1013946 (PMC12974954; doi:10.1371/journal.pcbi.1013946)
Supplement: S8 Fig — (PDF) [file pcbi.1013946.s009.pdf]

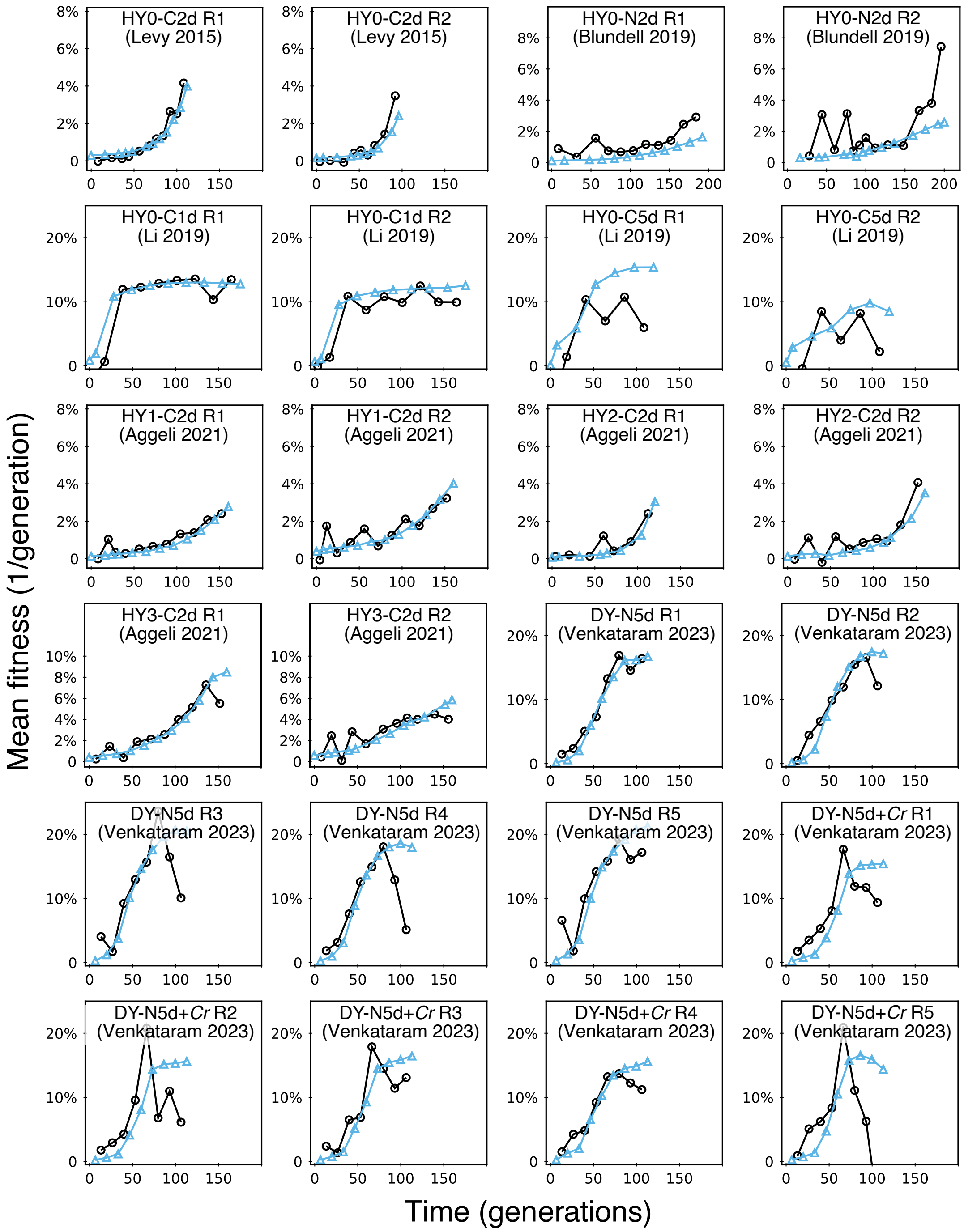

Figure S8. Mean fitness trajectories in published BLT datasets. Each panel represents a replicate of a BLT experiment, as indicated. Black lines show trajectories inferred by BASIL; blue lines show trajectories calculated from lineages identified as adapted.
