# Supplementary material for: Putting BASIL in a BLT: A Bayesian filtering method for estimating the fitness effects of nascent adaptive mutations
Source: PLoS Comput Biol. 2026 Feb 27;22(2):e1013946. doi: 10.1371/journal.pcbi.1013946 (PMC12974954; doi:10.1371/journal.pcbi.1013946)
Supplement: S9 Fig — (PDF) [file pcbi.1013946.s010.pdf]

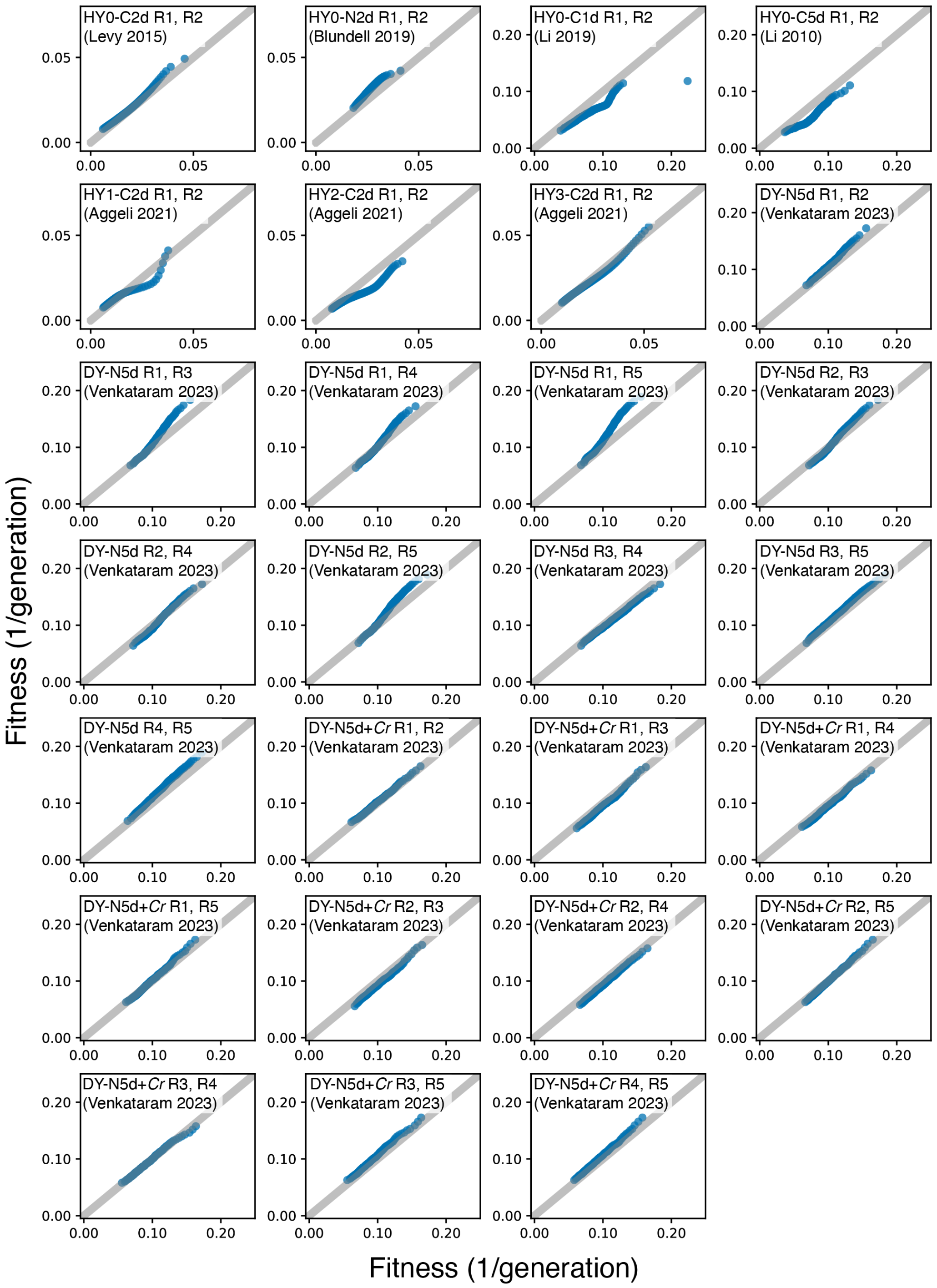

Figure S9. Quantile-quantile plots for the measured distributions of fitness effects (mDFEs) inferred across replicates. Each panel represents a pair of replicates from the same BLT study, as indicated. 100 quantiles are shown in each panel.
