# Supplementary material for: Putting BASIL in a BLT: A Bayesian filtering method for estimating the fitness effects of nascent adaptive mutations
Source: PLoS Comput Biol. 2026 Feb 27;22(2):e1013946. doi: 10.1371/journal.pcbi.1013946 (PMC12974954; doi:10.1371/journal.pcbi.1013946)
Supplement: S1 Table — (PDF) [file pcbi.1013946.s012.pdf]

	Linear ($\epsilon = 0$)		Quadratic ($\epsilon \neq 0$)	
	Poisson ($a = 1$)	Lin. 1 –	Quad. 1 ($a = 1$)	Quad. 2 –
Fitted $\hat{a}$	–	4.34	–	1.97
Fitted $\hat{\epsilon}$	–	–	0.0024	0.0016
RSS[1]	173.6	85.2	55.1	48.3
R^2	0.77	0.89	0.93	0.94

Table S1. Model fitting for the relationship between read-count mean and variance. [1]Residual sum of squares.
