# Supplementary material for: Putting BASIL in a BLT: A Bayesian filtering method for estimating the fitness effects of nascent adaptive mutations
Source: PLoS Comput Biol. 2026 Feb 27;22(2):e1013946. doi: 10.1371/journal.pcbi.1013946 (PMC12974954; doi:10.1371/journal.pcbi.1013946)
Supplement: S2 Table — (PDF) [file pcbi.1013946.s013.pdf]

Null model			Alternative model				
Name	p_{null}	RSS_{null}	Name	p_{alt}	RSS_{alt}	F	P-value
Poisson	0	173.6	Lin. 1	1	85.2	41.5	1.13×10^{-7}
Poisson	0	173.6	Quad. 1	1	55.1	50.7	1.63×10^{-11}
Poisson	0	173.6	Quad. 2	2	48.3	86.0	1.44×10^{-11}
Lin. 1	1	85.2	Quad. 2	2	48.3	29.9	2.87×10^{-6}
Quad. 1	1	55.1	Quad. 2	2	48.3	5.56	0.0235

Table S2. Goodness of fit comparison between models fitting the relationship between read-count mean and variance. In all cases $n = 41$.
