# Supplementary material for: Putting BASIL in a BLT: A Bayesian filtering method for estimating the fitness effects of nascent adaptive mutations
Source: PLoS Comput Biol. 2026 Feb 27;22(2):e1013946. doi: 10.1371/journal.pcbi.1013946 (PMC12974954; doi:10.1371/journal.pcbi.1013946)
Supplement: S3 Table — (PDF) [file pcbi.1013946.s014.pdf]

	Selection	
Parameter	Weak	Strong
$\langle s \rangle$	0.03	0.08
σ_s	0.01	0.015
$\langle N \rangle$	2.56×10^9	2.56×10^8
$\langle N_b \rangle$	10^7	10^6
D	256	256
R	10^7	10^6
σ_r^2	$6 \langle r_i \rangle$	$\langle r_i \rangle + 0.01 \langle r_i \rangle^2$

Table S3. Simulation parameters. For the weak selection regime, we chose the relationship between mean read count $\langle r_i \rangle$ and variance in read count σ_r^2 to be linear in order to make our simulations as similar to the work by Levy et al [3] as possible. For the strong selection regime, we chose the more realistic quadratic relationship, based on our results.
