# Supplementary material for: Putting BASIL in a BLT: A Bayesian filtering method for estimating the fitness effects of nascent adaptive mutations
Source: PLoS Comput Biol. 2026 Feb 27;22(2):e1013946. doi: 10.1371/journal.pcbi.1013946 (PMC12974954; doi:10.1371/journal.pcbi.1013946)
Supplement: S4 Table — (PDF) [file pcbi.1013946.s015.pdf]

		**BASIL**	**FitMut2**
Full data	# of adapt. lin.	60920	15386
Reduced coverage	# of adapt. lin.	49330	1131
	Change in calling rate	−19.0%	−98.1%
	Overlap	48738	1130
	Relative overlap	98.8%	99.9%
	s PCC[1]	95.8%	82.0%
	Error[2] in s	0.20%	0.83%
Reduced sampling	# of adapt. lin.	48206	55248
	Change in calling rate	−20.9%	+259.1%
	Overlap	48177	15381
	Relative overlap	99.9%	100, 0%
	s PCC[1]	97.9%	62.0%
	Error[2] in s	0.18%	1.29%

Table S4. Effects of down-sampling. The "Full" dataset is Replicate 1 of the HY0 strain in C-1d environment [3]. See Materials and Methods in the main text for details. [1]Pearson correlation coefficient. [2]Averaged absolute difference of s inferred in Full and Reduced data.
