# Supplementary material for: Putting BASIL in a BLT: A Bayesian filtering method for estimating the fitness effects of nascent adaptive mutations
Source: PLoS Comput Biol. 2026 Feb 27;22(2):e1013946. doi: 10.1371/journal.pcbi.1013946 (PMC12974954; doi:10.1371/journal.pcbi.1013946)
Supplement: S5 Table — (PDF) [file pcbi.1013946.s016.pdf]

Strain	Cond.	Ref.	Mean	Median	IQR	IDR
HY0	C-2d	[3]	0.12*	0.05*	0.02 (0.060)	0.11*
HY0	N-2d	[3]	0.48*	0.51*	0.15*	0.25*
HY0	C-1d	[29]	1.85*	1.74*	1.31*	0.72*
HY0	C-5d	[29]	2.01*	2.35*	0.31 (0.081)	0.15 (0.48)
HY1	C-2d	[32]	0.01*	0.11*	0.13*	0.79*
HY2	C-2d	[32]	0.41*	0.30*	0.60*	0.67*
HY3	C-2d	[32]	0.05*	0.05*	0.22*	0.15*
DY	N-5d	[33]	0.74*	0.66*	0.98*	1.49*
DY	N-5d+*Cr*	[33]	0.43*	0.43*	0.16 (0.070)	0.34*

Table S5. Differences between mDFE statistics across replicates. For each mDFE statistic, we report the absolute value of the pairwise difference in the statistic value, averaged across all pairs of replicates (see text for details).
* indicates P-value < 0.01 (permutation test); P-values ≥ 0.01 are shown in parentheses.
