# Supplementary material for: Putting BASIL in a BLT: A Bayesian filtering method for estimating the fitness effects of nascent adaptive mutations
Source: PLoS Comput Biol. 2026 Feb 27;22(2):e1013946. doi: 10.1371/journal.pcbi.1013946 (PMC12974954; doi:10.1371/journal.pcbi.1013946)
Supplement: Attachment — Submitted filename: PCOMPBIOL-D-25-00612_review.pdf [file pcbi.1013946.s018.pdf]

Kuo & Kryazhimskiy

Wednesday, April 23, 2025 12:06 PM

The manuscript tackles the problem of estimating lineage fitness in barcoded experiments. Specifically, it identifies an issue with what is arguably the current standard, explain this issue very well; and builds a principled alternative.

Strengths: This question is important; the authors' Bayesian approach unquestionably has merit & potential to do better, and building a better computational method is very timely, since with the current sequencing capabilities we are already in the regime where in many cases the accuracy is limited by faults in data processing, rather than data quality.

Separately, the paper is very well presented. It is written with beautiful clarity - quite a rarity - and explains everything remarkably well. So reading it was genuinely an enjoyable experience, and I applaud the authors for that, as it made the reviewing assignment much less of a chore, and much more about engaging with an interesting question and a proposed solution.

Main weaknesses: As the manuscript currently stands, it failed to convince me that the current implementation actually works as well as it should. The manuscript completely convinced me about the issue with the current method (Levy-Blundell; LB); and I am also quite certain that a Bayesian approach is a better alternative in principle. But the validation is unconvincing. Or more specifically: I think it fails to convincingly show a practical advantage (see below); but succeeds very clearly at showing a failure mode which - in practice - would almost certainly dissuade the practitioners from switching their approach. Which would be very unfortunate, since I genuinely do believe that the approach like that taken by the authors *should* be able to do much better than current state of the art.

Let me be clear: I believe that even if this cannot be remedied, the paper should still be published. Even if its own proposed solution isn't quite there, it is *mostly* there, and will serve as the foundation for someone else to build the actual answer. However, who would be best positioned to do this, if not the present authors? So I lay out the critique below in the hope that these issues are actually fixable in the present version already, as it would make the paper much more impactful. But if the authors tell me these fixes aren't actually feasible in the present scope, I will still support publication.

Major issues

Validation.
The issue:

> The authors' method works spectacularly on simulated data. However, these data are generated with the very same model that the Bayesian approach uses as its forward model. So while this is a very important validation to have, this alone is not compelling. The authors have to convince us that their approach works better than the current practice on real data. Without this, the authors' claim (line 399) is missing a very important caveat: "Thus, taken together, our results suggest that BASIL is a statistically robust, accurate and precise approach for the analysis *of BLT data*" (emphasis added). Not really! Should be, of SIMULATED BLT data. And for real data, the validation is very weak. Similarly: ""
>
> Specifically, the authors' application of BASIL to real data is limited to the discussion of the inferred trajectory of mean fitness. They claim "BASIL is overall successful" (line 418), but by what metric? There is no reference for comparison, so the only thing they assess is whether the mean fitness is "reproducible" across replicates, and whether it increases over time. Even if both were verified perfectly, these are extremely coarse statements. In practice, the claimed reproducibility

across replicates is approximate at best. As for mean fitness increasing, the only thing it could show convincingly would be a failure of the method (if the fitness fails to increase). And it does, in fact, show that failure occurring in two of 9 examples.

The authors are honest and transparent about this. But to present their method as a viable alternative to LB, they must have some compelling data-based demonstration of **some** clear gain in accuracy - at least in some regime. Then, as a practitioner, I could weigh this demonstrated improvement against the risks (a method with more moving parts/parameters; very long computation time; a demonstrated risk of a catastrophic failure due to secondary mutations). Without this, I have all those risks on one hand, and no obvious benefit on the other.

This is especially disappointing with respect to secondary mutations. This is *the* aspect where I would expect a Bayesian approach to be obviously superior to LB. So the inability of the present implementation to deal with that is rather heartbreaking.

Proposed revisions:

A. If the issue with declining mean fitness arises for the reason described in line 432, this feels like it should be solvable.
   - Thought 1: While I understand the rationale for the convenience of inferring lineage fitnesses and mean fitness separately, they are coupled. In particular, it is mathematically inconsistent for mean fitness to decline; and in fact, I know more: if I have a probability distribution for everyone's abundance and fitness at time k-1, I have a pretty good estimate of the expectation for mean s at time k (with some quantifiable uncertainty due to unobserved lineages, but their influence is bounded since their abundance is small). Thus, it feels like the issue described in line 432 is something that a Bayesian approach should be equipped to handle.
   - Thought 2: Secondary mutations. For a given lineage, uncertainty about fitness narrows over time. Then, new evidence starts contradicting this. Detecting this contradiction, and recognizing this as evidence of a mutation, seems like precisely the place where a Bayesian method should shine. If I have a small prior on the hypothesis, "this lineage mutated", at some point the new information makes this the more likely explanation, at which point you would reset the belief back to something wide and label this as a new lineage. Is this possible?

B. Separately, I believe the downsampling analysis could/should be expanded. We do not have a ground truth agasint which to compare the fitness values. However, if we treat the fitness values inferred from the full dataset as our best approximation of the method's ground truth, and then one could imagine comparing the robustness of the two methods as the dataset is reduced. The current Fig. 8 performs a related analysis, but it just shows that BASIL "works well". What the paper is missing is a compelling argument that BASIL is better than LB at something specific (in real data), and this analysis could provide that.

C. Finally, I spent some time reflecting on line 356: to determine fitness, the method picks the time when the belief distribution was most narrow. What is the mental model I should have for why this time would be at an intermediate timepoint rather than the end? Is this coupled to lineage abundance dynamics, such that fitness estimate keeps improving as the lineage rises, but eventually gets outcompeted by even fitter variants and the width of the estimate starts drifting?

   I'm confused here because naively, I keep adding data, and my Bayesian estimates should be most reliable once I included all the information. Once the abundance drops back into the noise, future updates are not improving my estimate. But it feels like they shouldn't spoil them either: New evidence should only widen the distribution if it materially contradicts the previously established belief. If it does contradict, this widening is relevant and should not be discarded just because we

had a moment of happy ignorance where we were sure in our beliefs (which later contradicted the observation). Is this a "plug" to deal with secondary mutations?

I would like to see some comment on this, and a diagnostic plot showing the dynamics of the width of the fitness distribution over time, for many lineages, as a function of their estimated abundance at that time. Perhaps these could be shown as trajectories on the [n vs width of s] plane, or in whichever way would be most interpretable. The current figure S4 has some of this information, but only shows a few examples rather than a systematic analysis, and it's hard to see where the width of the s distribution is most narrow.

Medium issues

Details of the underlying simulation model (line 119).

I don't think you can just refer to the SI here. A lot of the information about how it's structured is critical, and should not be stashed away in a separate document - especially the implementation of noise, whether the growth/"passaging" steps are explicitly modeled, etc. Please add a few sentences with a brief description of the salient parts.

The noise parameter epsilon --> I was very confused how it's treated.

- Is this a free parameter estimated at each iteration?
- Is it fixed to some set value, which the user is expected to adjusted for their dataset?
- Is it fixed once and for all?

Throughout the paper, this parameter is defined either with (line 341, 342, 347) or without (line 305, 327, 349, table S4) subscript k, making it unclear if its value changes with iterations. The phrasing of line 304 could be interpreted as saying it is a global parameter whose value is estimated to be the specified constant (based on the calibration experiment), but this is contradicted by later text. If this parameter is adjusted throughout the iterations, I'd recommend stating explicitly that the calibration experiment was meant to determined the appropriate family of noise models (indexed by epsilon), not *the* noise model.

Presentation of Fig. 2

I personally found that the discussion of the details of Fig. 2B and D to be more confusing rather than helpful. And delays the (much more useful) discussion of ***why*** the LB assumption of exact proportionality isn't right.

My suggestion - streamline the two paragraphs line 169-201 to get to the meat quicker. On first read, the actually useful part is lines 169-179 and 191-201: "The LB assumption isnt' exactly right. This is true both in simulations and in data. While the deviation looks small, it turns out to be sufficient to lead to errors. So let's understand where it's coming from." Before we understand where this is coming from, for me personally the discussion of lines 179-190 (how the error is affected by the abundance of chosen reference reads etc.) went completely above my head. I'm willing to believe that the small intercept **is enough to cause** estimation error; but the rest of the paragraph makes it sound like I'm expected to actually this relationship in detail already at this stage, but it's not really explained (and I don't think it's critical for the narrative).

The explanation of nonzero intercept (line 212 onwards):

The main point is that the estimating the true abundance based on read counts is a biased estimator, especially on the low end of sequence counts. I agree, and the authors mostly did a great job explaining this. But their choice of illustrating this with the case of n_(k-1)=0 is actually a bit misleading, because specifically zero is special.

As the authors correctly say later, this effect depends on the underlying distribution of lineages. If I had 10^9 lineages and only 10^6 reads, the authors' curve would actually be going way **under** 1:1

line. The one *exception* is for the point n_{k-1}=0, since the average n_k of such lineages, however small, can only be positive. So it seems to me that using the zero point is slightly misleading/oversimplifying. I understand the desire to explain the effect simply, so this is not a major point. But perhaps two simple examples with all lineages being neutral and equal abundances would illustrate this regression to the mean just as well while avoiding the issue of zero counts being special. (Equal abundances that should have resulted in 20 counts --> observing 1 is a rare fluke and the curve will be above 1:1.)

Use of term "evolution"

The word "evolution" in Figure 3, its legend, line 239, and line 253 should be replaced by "population dynamics" or something similar. Unless the proposed revision A.2 is implemented, in the present version, this model explicitly omits the possibility of a mutation, so calling this "evolution" can set up precisely the opposite expectation.

Notation

I found equation (3) and similar very difficult to read. First, everything seems like an index, whereas in fact b, m and pr are labels, and only i and k are indices. I'd recommend renaming "pr" into "prior", b -> "belief" and m-> "meas" (for measurement), all in roman typeface, to distinguish from math cursive of i and k. Also, I think specifying the left-hand side as being P_ik^b(n,s | r_k) (updated belief *given the data*) would make the Bayes theorem easier to recognize and the argument easier to follow.

Minor issues

"Lineage abundance that maximizes accuracy" - this idea appeared in two places (line 189, line 230). This may be a taste thing, but I feel like it's a misleading way of putting it. If some assumption is faulty and there is a systematic bias, which can cause is to overestimate fitness or underestimate fitness, then there is a crossover where the error would accidentally be zero. But I'm uncomfortable with framing this as saying that there is some magical correct abundance of lineages that would have given the right estimate, if only we knew what that abundance was, but "sadly that's difficult to determine". There isn't any good abundance. Or am I wrong? To me it sounded like the broken clock showing the right time twice a day, but sadly it's difficult to determine when the optimal time to look is.

Fig. 1: Including a legend could be helpful, and this figure could perhaps be made more black & white printing-friendly, if the points in panels BDFG differed in marker type and not just color.

Fig. 2 B, D - plotting error as "true minus inferred": I found this choice of sign very counterintuitive. Why this choice? Is this standard? Usually, the error of estimation is defined as estimated minus actual.

Table 2 - what is the purpose of this table in the main text? It takes up an entire page and (in my opinion) serves almost no purpose. Perhaps this should be in the SI.

Similarly, what is the usefulness of yellow lines as "reference" on Fig. 6? What does this comparison tell us?
