# Supplementary material for: Putting BASIL in a BLT: A Bayesian filtering method for estimating the fitness effects of nascent adaptive mutations
Source: PLoS Comput Biol. 2026 Feb 27;22(2):e1013946. doi: 10.1371/journal.pcbi.1013946 (PMC12974954; doi:10.1371/journal.pcbi.1013946)
Supplement: Attachment — Submitted filename: Reviewer Response.pdf [file pcbi.1013946.s019.pdf]

We thank the reviewers for carefully reading our manuscript and for their thoughtful and constructive comments. For the ease of cross-referencing, we divided the reviewers' comments into numbered sections. Before responding to their comments, we would like to draw the attention of reviewers to two major changes that we made.

1. Reviewer 1 asked for a direct comparison between BASIL and the LB method on real data. In the original manuscript, we have implemented a simplified version of the LB method for the purposes of dissecting its failure modes on simulated data. Our simplified implementation may not work as well on real data as the full implementation developed by Levy et al. The original LB method was recently superseded by FitMut2 (Ref. 43). Thus, we felt that it would be more useful and fair to compare BASIL to FitMut2. Our analysis shows that BASIL is superior to FitMut2.
2. We restructured the paper to accommodate multiple comments of both reviewers. Most importantly, we moved the down-sampling analysis up, so that former Figure 8 is now Figure 6. We also removed former Figure 7 (since it is essentially redundant with Figure S10) and we moved the large former Table 2 to Data S1, Tab 3.

Reviewer 1

Summary

The manuscript tackles the problem of estimating lineage fitness in barcoded experiments. Specifically, it identifies an issue with what is arguably the current standard, explain this issue very well; and builds a principled alternative.

Strengths: This question is important; the authors' Bayesian approach unquestionably has merit & potential to do better, and building a better computational method is very timely, since with the current sequencing capabilities we are already in the regime where in many cases the accuracy is limited by faults in data processing, rather than data quality.

Separately, the paper is very well presented. It is written with beautiful clarity - quite a rarity - and explains everything remarkably well. So reading it was genuinely an enjoyable experience, and I applaud the authors for that, as it made the reviewing assignment much less of a chore, and much more about engaging with an interesting question and a proposed solution.

Main weaknesses: As the manuscript currently stands, it failed to convince me that the current implementation actually works as well as it should. The manuscript completely convinced me about the issue with the current method (Levy-Blundell; LB); and I am also quite certain that a Bayesian approach is a better alternative in principle. But the validation is unconvincing. Or more specifically: I think it fails to convincingly show a practical advantage (see below); but succeeds very clearly at showing a failure mode which - in practice - would almost certainly dissuade the practitioners from switching their approach. Which would be very unfortunate, since I genuinely do believe that the

approach like that taken by the authors *should* be able to do much better than current state of the art.

Let me be clear: I believe that even if this cannot be remedied, the paper should still be published. Even if its own proposed solution isn't quite there, it is *mostly* there, and will serve as the foundation for someone else to build the actual answer. However, who would be best positioned to do this, if not the present authors? So I lay out the critique below in the hope that these issues are actually fixable in the present version already, as it would make the paper much more impactful. But if the authors tell me these fixes aren't actually feasible in the present scope, I will still support publication.

Author response. We are pleased that the reviewer found merit in our manuscript. We address his specific critiques below.

Reviewer comment 1.1

Major issues

Validation:

The authors' method works spectacularly on simulated data. However, these data are generated with the very same model that the Bayesian approach uses as its forward model. So while this is a very important validation to have, this alone is not compelling. The authors have to convince us that their approach works better than the current practice on real data. Without this, the authors' claim (line 399) is missing a very important caveat: "Thus, taken together, our results suggest that BASIL is a statistically robust, accurate and precise approach for the analysis *of BLT data*" (emphasis added). Not really! Should be, of SIMULATED BLT data. And for real data, the validation is very weak. Similarly: ""

Specifically, the authors' application of BASIL to real data is limited to the discussion of the inferred trajectory of mean fitness. They claim "BASIL is overall successful" (line 418), but by what metric? There is no reference for comparison, so the only thing they assess is whether the mean fitness is "reproducible" across replicates, and whether it increases over time. Even if both were verified perfectly, these are extremely coarse statements. In practice, the claimed reproducibility across replicates is approximate at best. As for mean fitness increasing, the only thing it could show convincingly would be a failure of the method (if the fitness fails to increase). And it does, in fact, show that failure occurring in two of 9 examples.

Author response. We agree with the reviewer that the main support for the superior performance of BASIL over the LB method comes from simulated data. We reasoned that if a method fails on simulated data, it is very unlikely that it would perform better on real data. We also agree that obtaining additional evidence of improved performance on real data would further strengthen our results. To address this issue, we carried out two additional analyses.

First, as suggested by the reviewer below, we directly compared how BASIL and FitMut2 behave as we down-sample the Levy et al data. Please see Reviewer

Comment 1.5 and our response there. Second, we applied BASIL and FitMut2 to the BLT data by Venkataram et al (Ref. 33). In that paper, we used bulk competition assays to independently measure the fitness of 410 clones isolated from BLT experiments. Although clone fitness obtained in these assays may differ somewhat from the fitness of its parent lineage in the BLT experiment, these measurements are as close to the ground truth as it is currently possible. Thus, we compared fitness estimates for adapted lineages obtained from BLT data by both methods to those estimated in competition assays. The results are presented in LL 446–452 and in Figure S9. The bottom line that BASIL dramatically outperforms FitMut2 in both of these analyses.

Reviewer comment 1.2

The authors are honest and transparent about this. But to present their method as a viable alternative to LB, they must have some compelling data-based demonstration of some clear gain in accuracy - at least in some regime. Then, as a practitioner, I could weigh this demonstrated improvement against the risks (a method with more moving parts/parameters; very long computation time; a demonstrated risk of a catastrophic failure due to secondary mutations). Without this, I have all those risks on one hand, and no obvious benefit on the other.

Author response. Given the results of the new down-sampling analysis (see our response to Reviewer comment 1.5), the side-by-side comparison with competition assay data (see our response to Reviewer comment 1.1), and the fact that, in our view, declines in mean fitness do not actually indicate a catastrophic failure (see our response to Reviewer comment 1.3), we hope the reviewer agrees that the case for using BASIL is now quite strong.

Reviewer comment 1.3

This is especially disappointing with respect to secondary mutations. This is *the* aspect where I would expect a Bayesian approach to be obviously superior to LB. So the inability of the present implementation to deal with that is rather heartbreaking.

Author response. We disagree with the reviewer's assessment of the severity of this problem, for the following reason. The ultimate purpose for which the BLT method was designed is twofold: (i) to estimate the DFE, i.e., the distribution of fitness effects of **single-mutant** neighbors of a given "wildtype" genotype and (ii) sample many adapted mutants from this neighborhood so that their properties can be investigated further (see e.g., Refs. 3, 32, 33). In other words, the key point is to capture lineages while they carry **single** driver mutations and **before** they acquire secondary mutations (see Refs. 3 and https://doi.org/10.1016/j.ygeno.2014.09.005). Once secondary mutations become prevalent, researchers generally stop their BLT experiment because the yield of useful information drops dramatically. This is the reason for why BLT experiments typically last for only 100 to 200 generations.

It is of course important in general to understand the properties of secondary mutations. However, the BLT experiment has little power to do so because by the time

secondary mutations become prevalent much of barcode diversity had already been lost, so that a typical barcoded lineage contains a multitude of adapted genotypes. To resolve secondary mutations, other approaches, such as “rebarcoding”, must be employed (see Refs. 5 and 32).

Given the main purpose of the BLT experiment, we do not see declines in mean fitness estimates caused by secondary mutations as a major problem. Instead, such declines can be viewed as a signal that the data at later time points should not be used.

In response to this comment, we added clarifications to the text at LL. 498–519.

Reviewer comment 1.4

Proposed revisions:

A. If the issue with declining mean fitness arises for the reason described in line 432, this feels like it should be solvable.

Author response. We agree with the reviewer that this issue is most likely solvable, and both of reviewer’s suggestions below are very insightful. However, as discussed above (see our response to Reviewer comment 1.3), we think that this issue is not as serious as the reviewer suggests, at least for the question that we are addressing. Furthermore, implementing these suggestions is quite challenging, as we explain below. Therefore, we decided not to implement them in the current version of BASIL, but we think these are good directions for future improvements.

○ Thought 1: While I understand the rationale for the convenience of inferring lineage fitnesses and mean fitness separately, they are coupled. In particular, it is mathematically inconsistent for mean fitness to decline; and in fact, I know more: if I have a probability distribution for everyone's abundance and fitness at time k-1, I have a pretty good estimate of the expectation for mean s at time k (with some quantifiable uncertainty due to unobserved lineages, but their influence is bounded since their abundance is small). Thus, it feels like the issue described in line 432 is something that a Bayesian approach should be equipped to handle.

Author response. The reviewer is absolutely correct that our approach is not fully Bayesian because of how we estimate mean fitness. The reviewer’s proposed approach is a move towards a “more Bayesian” version but it still not fully Bayesian because, as we understand it, the inference of mean fitness and the inference of lineage fitnesses would still occur into two steps. As long as these inferences are done separately, whether within the maximum likelihood or a Bayesian framework, we think that pathologies may still occur. To avoid them, one ought to implement a fully Bayesian approach. However, since mean fitness and lineage fitnesses are obviously correlated, we would have to simultaneously estimate **the entire joint distribution of all lineage fitnesses and the mean fitness** at each time step. Naively, doing so for ~10^5 lineages would seem impossible. However, this joint distribution should have a very sparse correlation structure, i.e., lineage fitnesses presumably should be directly correlated only with mean fitness but not with each other. Thus, we think that

estimating this joint distribution should be possible in principle. But how to do this in practice is not entirely clear to us at the moment and would require additional research. This work would go far beyond the scope of this paper, but we think it is a very interesting direction for future research.

○ Thought 2: Secondary mutations. For a given lineage, uncertainty about fitness narrows over time. Then, new evidence starts contradicting this. Detecting this contradiction, and recognizing this as evidence of a mutation, seems like precisely the place where a Bayesian method should shine. If I have a small prior on the hypothesis, "this lineage mutated", at some point the new information makes this the more likely explanation, at which point you would reset the belief back to something wide and label this as a new lineage. Is this possible?

Author response. This is a very good suggestion, and it is at the top of the list of the next improvements. However, we are hesitant to implement it immediately because of the following complication. How should we treat the parameter that controls the probability of a new beneficial mutation within a lineage? We could treat it as a fixed meta-parameter, which is what the reviewer seems to suggest. However, presumably, this probability can vary substantially between experiments, and it is not obvious how the user should go about choosing this parameter. Alternatively, we could treat this mutational probability as a regular parameter within the Bayesian framework and estimate it from data. We think this is the right approach, but it would require us to reformulate our model. It is definitely doable, but given the effort it would require, we think it makes more sense to implement in the next version of BASIL.

Reviewer comment 1.5

B. Separately, I believe the downsampling analysis could/should be expanded. We do not have a ground truth agasint which to compare the fitness values. However, if we treat the fitness values inferred from the full dataset as our best approximation of the method's ground truth, and then one could imagine comparing the robustness of the two methods as the dataset is reduced. The current Fig. 8 performs a related analysis, but it just shows that BASIL "works well". What the paper is missing is a compelling argument that BASIL is better than LB at something specific (in real data), and this analysis could provide that.

Author response. We have carried out the down-sampling analysis with FitMut2 on the Levy et al dataset (this is the largest and “cleanest” dataset to date). The results are now presented in the new Figure 6 and discussed in LL 453–490. We find that, while BASIL is quite robust with respect to data reduction of this magnitude, FitMut2 experiences a major loss of statistical power upon a reduction of coverage and a catastrophic increase in the rate of false positives upon a reduction in sampling frequency.

Reviewer comment 1.6

C. Finally, I spent some time reflecting on line 356: to determine fitness, the method picks the time when the belief distribution was most narrow. What is the mental model I should have for why this time would be at an intermediate timepoint rather than the end? Is this coupled to lineage abundance dynamics, such that fitness estimate keeps improving as the lineage rises, but eventually gets outcompeted by even fitter variants and the width of the estimate starts drifting?

I'm confused here because naively, I keep adding data, and my Bayesian estimates should be most reliable once I included all the information. Once the abundance drops back into the noise, future updates are not improving my estimate. But it feels like they shouldn't spoil them either: New evidence should only widen the distribution if it materially contradicts the previously established belief. If it does contradict, this widening is relevant and should not be discarded just because we had a moment of happy ignorance where we were sure in our beliefs (which later contradicted the observation). Is this a "plug" to deal with secondary mutations?

I would like to see some comment on this, and a diagnostic plot showing the dynamics of the width of the fitness distribution over time, for many lineages, as a function of their estimated abundance at that time. Perhaps these could be shown as trajectories on the [n vs width of s] plane, or in whichever way would be most interpretable. The current figure S4 has some of this information, but only shows a few examples rather than a systematic analysis, and it's hard to see where the width of the s distribution is most narrow.

Author response. This is a very good point. The motivation for selecting the time point with the lowest posterior variance was to avoid the potential complications associated with secondary mutations, exactly as the reviewer discerned. To address the reviewer's concern, we examined which time point is actually being picked by BASIL in all of the datasets. The results of this analysis are now provided in Data S1, Tab 3 (columns R–U). We found that, across all studies,

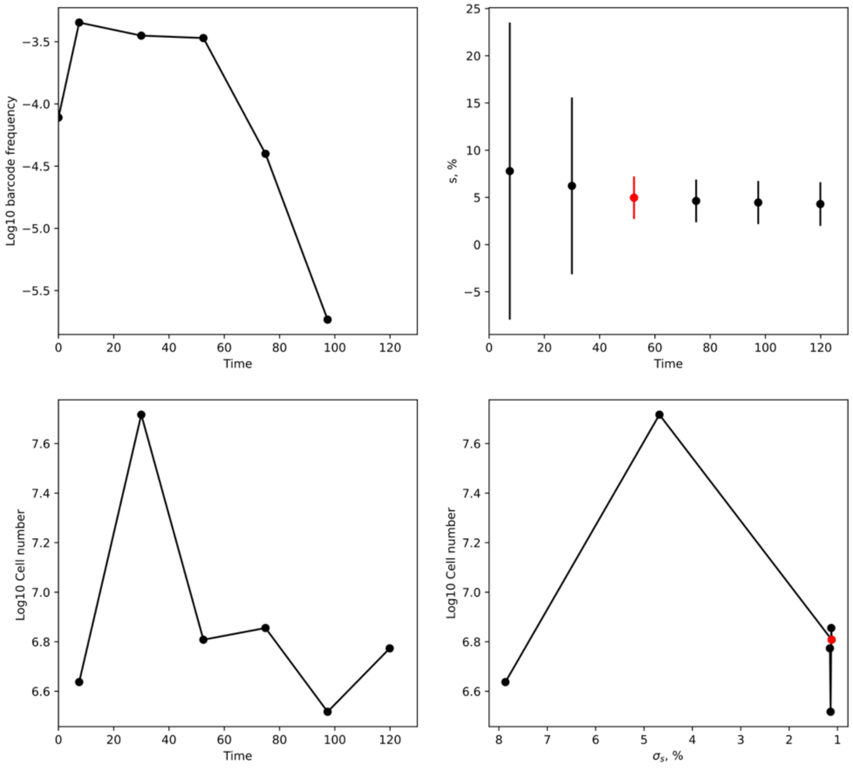

Figure 1. Lineage 7864 from Li et al (2019), Evo5D, Replicate 2. Red point highlights the time point with the lowest variance of the posterior distribution.

BASIL picks the last time point for the final fitness estimation for the average of 98.76% of lineages. In most cases, the fraction is even higher, exceeding 99%. The second most commonly picked time point is the one before the last one, which leaves on average 0.09% of lineages for which an earlier time point is picked. We manually examined the frequency trajectories and our estimates for many of these latter lineages (see Figure 1, which shows one the worst cases). We found that, in all cases, the posterior distribution in fact changes very little between the time point that is picked and the last time point. In other words, it appears that BASIL occasionally picks an intermediate time point because of small fluctuations in the estimation uncertainty, which does not represent a serious cause for concern.

We have added a discussion of this issue to LL. 381–392.

Reviewer comment 1.7

Medium issues

Details of the underlying simulation model (line 119).

I don't think you can just refer to the SI here. A lot of the information about how it's structured is critical, and should not be stashed away in a separate document - especially the implementation of noise, whether the growth/"passaging" steps are explicitly modeled, etc. Please add a few sentences with a brief description of the salient parts.

Author response. Done.

Reviewer comment 1.8

The noise parameter epsilon --> I was very confused how it's treated.

- Is this a free parameter estimated at each iteration?
- Is it fixed to some set value, which the user is expected to adjusted for their dataset?
- Is it fixed once and for all?

Throughout the paper, this parameter is defined either with (line 341, 342, 347) or without (line 305, 327, 349, table S4) subscript k, making it unclear if its value changes with iterations. The phrasing of line 304 could be interpreted as saying it is a global parameter whose value is estimated to be the specified constant (based on the calibration experiment), but this is contradicted by later text. If this parameter is adjusted throughout the iterations, I'd recommend stating explicitly that the calibration experiment was meant to determined the appropriate family of noise models (indexed by epsilon), not *the* noise model.

Author response. Thank you for pointing out this confusing issue. Epsilon is a nuisance parameter, and we tried various ways of fitting it. In the end, we settled on using it as a free parameter at each time interval along with mean fitness. We have now

revised the main text and the supplementary material to clarify this issue, as suggested, in particular in LL. 332–343. We left ϵ without an index in equation in L. 378 because it simply serves as a placeholder in the argument of the likelihood function (similarly, $\bar{s}$ also lacks an index in this equation).

Reviewer comment 1.9

Presentation of Fig. 2

I personally found that the discussion of the details of Fig. 2B and D to be more confusing rather than helpful. And delays the (much more useful) discussion of *why* the LB assumption of exact proportionality isn't right.

My suggestion - streamline the two paragraphs line 169-201 to get to the meat quicker. On first read, the actually useful part is lines 169-179 and 191-201: "The LB assumption isnt' exactly right. This is true both in simulations and in data. While the deviation looks small, it turns out to be sufficient to lead to errors. So let's understand where it's coming from." Before we understand where this is coming from, for me personally the discussion of lines 179-190 (how the error is affected by the abundance of chosen reference reads etc.) went completely above my head. I'm willing to believe that the small intercept is enough to cause estimation error; but the rest of the paragraph makes it sound like I'm expected to actually this relationship in detail already at this stage, but it's not really explained (and I don't think it's critical for the narrative).

Author response. Following reviewer's suggestion, we somewhat reorganized this now new section "Statistical causes for bias of the LB method" and removed some unnecessary verbiage in this paragraph (LL 204–251). However, we think that, *regardless of the reasons why the intercept is non-zero,* it is important for the reader to understand how such intercept can affect mean fitness estimation and subsequently bias the estimation of selection coefficients of all adapted lineages. We therefore left this discussion in the text, with some clarifying edits.

Reviewer comment 1.10

The explanation of nonzero intercept (line 212 onwards):

The main point is that the estimating the true abundance based on read counts is a biased estimator, especially on the low end of sequence counts. I agree, and the authors mostly did a great job explaining this. But their choice of illustrating this with the case of n_(k-1)=0 is actually a bit misleading, because specifically zero is special.

As the authors correctly say later, this effect depends on the underlying distribution of lineages. If I had 10^9 lineages and only 10^6 reads, the authors' curve would actually be going way under 1:1 line. The one *exception* is for the point n_{k-1}=0, since the average n_k of such lineages, however small, can only be positive. So it seems to me that using the zero point is slightly misleading/oversimplifying. I understand the desire to explain the effect simply, so this is not a major point. But perhaps two simple

examples with all lineages being neutral and equal abundances would illustrate this regression to the mean just as well while avoiding the issue of zero counts being special. (Equal abundances that should have resulted in 20 counts --> observing 1 is a rare fluke and the curve will be above 1:1.)

Author response. Thank you for this excellent suggestion. This explanation is much better than ours. We updated the text as suggested (LL. 232–251).

Reviewer comment 1.11

Use of term "evolution"

The word "evolution" in Figure 3, its legend, line 239, and line 253 should be replaced by "population dynamics" or something similar. Unless the proposed revision A.2 is implemented, in the present version, this model explicitly omits the possibility of a mutation, so calling this "evolution" can set up precisely the opposite expectation.

Author response. Although the distinction between "population dynamics" and "evolution" is admittedly fuzzy, changes in frequencies of pre-existing alleles are considered to be a canonical example of evolution (see for example, https://doi.org/10.1016/j.tree.2007.09.008). So, we prefer to stick with this terminology, but we clarified what we mean in (LL. 140–154).

Reviewer comment 1.12

Notation

I found equation (3) and similar very difficult to read. First, everything seems like an index, whereas in fact b, m and pr are labels, and only i and k are indices. I'd recommend renaming "pr" into "prior", b -> "belief" and m-> "meas" (for measurement), all in roman typeface, to distinguish from math cursive of i and k. Also, I think specifying the left-hand side as being P_ik^b(n,s | r_k) (updated belief *given the data*) would make the Bayes theorem easier to recognize and the argument easier to follow.

Author response. We changed most of the notations as suggested, but we left $P_{ik}^{\mathrm{belief}}(n,s)$ in equation (3) without the conditioning, for the sake of consistency with the previous use of $P_{ik-1}^{\mathrm{belief}}(n,s)$ and the brevity of notations. We provide the full notations in Table S5.

Reviewer comment 1.13

Minor issues

"Lineage abundance that maximizes accuracy" - this idea appeared in two places (line 189, line 230). This may be a taste thing, but I feel like it's a misleading way of putting it. If some assumption is faulty and there is a systematic bias, which can cause is to overestimate fitness or underestimate fitness, then there is a crossover where the error

would accidentally be zero. But I'm uncomfortable with framing this as saying that there is some magical correct abundance of lineages that would have given the right estimate, if only we knew what that abundance was, but "sadly that's difficult to determine". There isn't any good abundance. Or am I wrong? To me it sounded like the broken clock showing the right time twice a day, but sadly it's difficult to determine when the optimal time to look is.

Author response. We agree, and we have revised these paragraphs (now new Section “Statistical causes for bias of the LB method”).

Reviewer comment 1.14

Fig. 1: Including a legend could be helpful, and this figure could perhaps be made more black & white printing-friendly, if the points in panels BDFG differed in marker type and not just color.

Author response. Done.

Reviewer comment 1.15

Fig. 2 B, D - plotting error as "true minus inferred": I found this choice of sign very counterintuitive. Why this choice? Is this standard? Usually, the error of estimation is defined as estimated minus actual.

Author response. Changed as suggested.

Reviewer comment 1.16

Table 2 - what is the purpose of this table in the main text? It takes up an entire page and (in my opinion) serves almost no purpose. Perhaps this should be in the SI.

Author response. Upon reflection, we agree, and we moved this table to SI (now Data S1, Tab 3).

Reviewer comment 1.17

Similarly, what is the usefulness of yellow lines as "reference" on Fig. 6? What does this comparison tell us?

Author response. We decided to remove former Figure 6 since it is redundant with Figure S10. But we prefer to keep the analogous yellow curve in Figure 7, as some readers may be interested in comparing measured DFEs across conditions and strains.

Reviewer 2

Summary

Kuo and Kryazhimskiy critically evaluate an existing method (neutral decline or Levy-Blundell) for inferring fitness effects of spontaneous mutants from barcoded lineage tracking data, using simulated data and previously-published experimental data (consistently applying the same method across these data sets). Besides showing how the results depend on the average strength of selection and the crucial choice of putatively neutral lineages as a reference, they find that a major limitation of this method is errors in the inferred mean fitness, which leads to errors in all individual mutant fitnesses as well. They also make an interesting point that a barcode's read count is a biased estimator of its true frequency. Then they develop a new method called BASIL which uses an iterative Bayesian algorithm to infer mutant fitness from these data sets. The major advantage of BASIL seems to be that reference lineages for the mean fitness calculation do not need to be manually chosen. They apply BASIL to the same simulated and experimental data sets and find that it generally works much better than the older methods, especially for capturing the mean fitness under both weak and strong selection.

Altogether the paper is solid. The previous literature on these methods is highly technical and often confusing in my opinion, and having a well-written paper that critically and clearly addresses these methods (besides introducing a new and better one) is valuable. I only have a few minor comments and questions (some of which are my own curiosity). I separated these into "major" and "minor" just to highlight two comments that I think are a little more substantial, but none of these should be impediments to publication.

Author response. We appreciate the reviewer’s overall positive evaluation of our work.

Reviewer comment 2.1

Major comments

1. I understand that the authors tried to keep the main text streamlined by putting a lot of details in the supplement. But this being a technical methods paper, most readers are probably going to be people who want to implement these methods themselves, and thus will want to know those details, requiring them to flip back and forth a lot between the main text and the supplement. I would therefore suggest that the authors consider just making the main text longer and more technical so at least it can be read more linearly by its main readership. For example, Secs. 4 and 5 in the supplement are critical for anyone who wants to understand this paper, so some of that material could be moved to the main text.

Author response. We hope that this paper will be read not only by mathematically savvy computational biologists but also by less mathematically inclined potential users who just want to understand the essence of the method and apply it. So, we attempted to strike a balance between providing enough details to get the main ideas across but

also not overwhelming the latter type of readers. At the same time, we hope that the technically inclined readers will simply read Section 3 of the SI instead of respective section of the main text and a small amount of back-and-forth flipping will not deter them from too much. Having said that, in response to this comment and Reviewer comment 1.7, we moved large parts of the SI to the Methods section, expanded the description of key aspects of the method in the Results, and added some clarifications.

Reviewer comment 2.2

2. Could the authors say a little more about how to apply their method to other data sets? In particular, I am wondering what aspects of their method need to be adjusted for each new data set or experimental system and protocols, and if there are any experimental design choices that could be made in advance to accommodate them. (I realize this is not a best-practices paper for BLT experiments, but the data analysis method inevitably informs such considerations.) For example, the authors calibrate a model of how variance in read counts scales with mean read counts by sequencing a test sample with known barcode frequencies (Fig. 4). Is that something everyone needs to do first for their own system and protocols, or do they think their specific model and parameter values will hold across most cases? What about the prior distribution (with a normal distribution of fitness with standard deviation 0.1) and the hyperparameter beta? Is there some procedure we need to follow to calibrate them for our own data/systems?

Author response. Thank you for this excellent suggestion. We now provide additional implementation details in Section 5.5. of the SI. We hesitate to provide strong recommendations, but we extensively describe our own practices throughout the text (including some mentions in the Discussion). Briefly, we use a quadratic dependence of noise variance on the mean with ϵ as a fitting parameter, specific to each time interval in each dataset. We think that the choice of the prior distribution is not too important as long as it is sufficiently broad. Finally, we fixed the hyperparameter to be 3.3, but we think it would be worth revisiting this value on a broader set of simulated datasets, something that we hope to do in the future.

Reviewer comment 2.3

Minor comments

1. Line 9 "recently developed": I quibble with this characterization of BLT — the Levy et al. paper that started all this is over 10 years old now.

Author response. We removed “recently developed” from the abstract.

Reviewer comment 2.4

2. Line 15 "currently standard": Is the Levy-Blundell method actually that standard? I am not familiar with every study on BLT from the last 10 years, but I didn't think that everyone who does these experiments uses the same approach.

Author response. Perhaps "standard" is too strong. We have changed it to "commonly used".

Reviewer comment 2.5

3. Lines 43-44 "this strategy preferentially captures mutations with the strongest fitness benefits because they are least likely to be lost by genetic drift or clonal interference": How do the authors reconcile this statement with the argument in the first paragraph that clonal interference means one must know the whole distribution of beneficial mutants? I understand the authors' points here, but this latter statement seems to undercut the former. That is, if the same few mutants with strongest fitness effects consistently establish in an evolution experiment, then it seems like clonal interference isn't all that important and one doesn't need to know the whole DFE, at least if one just wants to predict evolutionary dynamics. On the other hand, if an evolution experiment yields many different established mutants, then clonal interference is significant but then one can get a decent sample of beneficial mutants just from established mutants in an evolution experiment. So doesn't that mean that high-resolution lineage tracking isn't crucial in either case? 'm playing devil's advocate here, but I'd like to know what the authors think about this.

Author response. We think Levy et al provided an excellent demonstration of the utility of high-resolution lineage tracking in Figure 4 of their 2015 *Nature* paper. We added a clarifying sentence to this paragraph to avoid this seeming contradiction (LL. 43–47).

Reviewer comment 2.6

4. Lines 131-132 "Low-abundance lineages are those represented by 20 to 40 reads, which corresponds to 20 to 40 cells": Doesn't this depend on the read depth and the number of cells in the sample used for sequencing (i.e., it assumes they are approximately the same)? Is that actually true for most of these experiments?

Author response. The fact that the number of reads coincides with the number of cells is true by coincidence in our simulations. The corresponding numbers for real data are provided in Data S1, Tab 3 (former Table 2).

Reviewer comment 2.7

5. Figure 1A: I noticed that the inferred mean fitness in this case is actually negative; can the authors comment on why that happens and what it means?

Author response. We thank the reviewer for drawing attention to this fact. This is another consequence of the incorrectness of the neutral decline equation (1), especially when low-abundance lineages are used to infer mean fitness (as in Figure 1A). In Figure 2A, we can see that the expected normalized read count $\tilde{r}_k = \bar{r}_k(R_{k-1}/R_k)$ at the next time point for lineages with the read count r_{k-1} at the previous time point depends on r_{k-1} linearly (at least at early time points) but with a non-zero intercept (dark gray lines). Due to this positive intercept, we have $\bar{r}_k/R_k > r_{k-1}/R_{k-1}$ for low-abundance lineages.

However, if we infer $\bar{s}_k$ using equation (1), which does not account for the intercept, the fact that $\bar{r}_k/R_k > r_{k-1}/R_{k-1}$ implies that $\bar{s}_k$ must be negative. We added a brief note on this point to the text (L 213).

Reviewer comment 2.8

6. Line 170: I think it would be clearer to denote this rescaled average read count as being for the time point $k - 1$ rather than k (i.e., denote as $\tilde{r}_{k - 1}$ rather than $\tilde{r}_k$), since the idea is that it estimates the expected read count at time $k - 1$ (with true value already denoted as $r_{k - 1}$, plotted in Fig. 2).

Author response. This seems incorrect. We define $\tilde{r}_k = \bar{r}_k(R_{k-1}/R_k)$. In other words, $\tilde{r}_k$ is just the average number of reads at the next time point t_k, averaged across all lineages whose read count was r_{k-1} at the previous time point t_{k-1}, adjusted to account for variation in coverage. We have now clarified this definition in LL. 187–192.

Reviewer comment 2.9

7. Figure 2E: One thing I noticed throughout this paper was how much cleaner the Levy et al. data seems to be compared to all the more recent data sets (see also Figs. 6 and 7). Do the authors know why that is?

Author response. Indeed, the Levy et al dataset appears to be the cleanest of all so far. Based on the examination of Data S1, Tab 3 (former Table 2), we suspect this could be due to a combination of factors: (*i*) they have the deepest coverage, exceeding 100 reads per lineage at the initial time point, but also (*ii*) among the largest numbers of lineages, both of which reduce sampling noise. (*iii*) Selection appears to be fairly weak, and (*iv*) their initial average lineage size is fairly large (~150), which implies that there are relatively few lineage extinctions, but not too large to cause problems with multiple mutations per lineages.

Reviewer comment 2.10

8. Lines 224-225 "it relies on the assumption that the measured barcode frequency is an unbiased estimator of the true lineage frequency in the population, which is in general incorrect": Can the authors say more about this? There was a very interesting section on the supplement about this (Sec. 4.3.2), but I didn't follow how it is (or isn't) related to the other issues with bias in the mean fitness and the shift in expected read counts for very low reads. In general some more explanation about to what extent these effects are all caused by the same underlying assumption, or are caused by different assumptions, would be helpful. My understanding is that there are two distinct issues, both arising from the use of Eq. 1. First is the choice of putatively neutral lineages as a reference, which is potentially arbitrary and can lead to biases. Second is the point mentioned here, which is that Eq. 1 assumes that a barcode's read frequency is an unbiased estimated of its true frequency.

Author response. Our wording was not entirely accurate here. For any given lineage, its barcode frequency is an unbiased estimator of its population frequency (see Figure 4A). In other words, if we measure the barcode frequency of a given lineage in replicate experiments (as we have done in Figure 4A), the average measured barcode frequency will be equal to the lineage's population frequency. But this is not what happens in the neutral decline method. There, we group together all lineages with the same barcode frequency at a given time point. The expected frequency of these lineages in the population is not equal to their barcode frequency because some lineages by chance got more reads than expected and some got less. We now provide a simple example of this problem in LL 232–251, thanks to Reviewer 1 (see Reviewer comment 1.10).

This inconsistency between the measured barcode frequency and the expected lineage frequency conditional on it is much worse for low-frequency lineages than for high-frequency lineages. This is why the choice of the neutral lineages is so important.

We have now revised this part of the text (now new section "Statistical causes for bias of the LB method"), which hopefully conveys this reasoning more clearly.

Reviewer comment 2.11

9. Lines 414-415 "on the basis of identified adapted lineages": What does this mean? Does it mean that some individual lineages were determined to be adapted independently of the BLT data (e.g., by isolating clones for them and performing separate competition experiments against the ancestor)?

Author response. Here, we can carry out a sanity check for BASIL performance by comparing its inferred mean fitness with the mean fitness calculated a posteriori, on the basis of lineages that BASIL identified as adapted. This is described in detail in Section 6.3 of the SI. These are two different (albeit not entirely independent) calculations. A substantial discrepancy between the results of these two calculations would suggest a problem in the inference. We changed the language here to clarify what we are doing here and why (LL. 498–500), and more details are provided in Section 6.3 of the SI.

Reviewer comment 2.12

10. Line 473 "for the inferring": Extraneous "the"

Author response. Fixed.

Reviewer comment 2.13

11. Supplement Sec. 2.3: This says there were 2.3e5 total reads across all 10 replicate sequencing samples (which seems quite small to me), but in the main text (line 285) it says 2e5 reads per replicate. Is one of these a typo, or do I misunderstand?

Author response. It should be $2.3{\times}10^5$ reads per replicate. We fixed it.

Reviewer comment 2.14

12. Supplement Sec. 2.4: The text here talks about 26 clones (unique barcodes?), but I don't understand how those were allocated across the target frequencies in the experimental setup from Sec. 2.2. That says there are supposed to be barcodes at six distinct frequencies in the test culture, so I was expecting six unique barcodes (or an integer multiple of that).

Author response. For each of 5 frequencies, we prepared 5 distinct barcodes, yielding 25 distinct barcodes. The remainder of the population carried an additional distinct barcode. We edited this section (now in Materials and Methods) for clarity.

Reviewer comment 2.15

13. Supplement Sec. 2.4, four lines above Eq. S2 "has its an unknown": Extraneous "its"

Author response. Fixed.

Reviewer comment 2.16

14. Supplement Sec. 4.1, Eq. S6: This model from Levy et al. always struck me as overkill, and I'd like to know what the authors of this paper think. To me it seems like the stochastic birth and death during a batch growth cycle is likely to be negligible compared to the stochasticity of the dilution step, and therefore unnecessary to model stochastically (rather than just as deterministic exponential growth). Indeed, that seems to be how the authors simulate it themselves here (Sec. 3), with deterministic growth between dilutions and Poisson sampling at the dilution. Is there something I'm missing?

Author response. The reviewer is correct that we model and simulate only stochasticity during the dilution step, with exponential growth being deterministic. We think that both choices are reasonable, but neither of them fully captures the complexity of growth even in the simplest conditions. We think that in the context of BLT data analysis, both choices are motivated mostly by practical considerations, such as the ability to obtain analytical results and computational speed.

Reviewer comment 2.17

15. Supplement Sec. 4.3.2, line above Eq. S11 "can also be re-write": Typo

Author response. Fixed.

Reviewer comment 2.18

16. Supplement Sec. 4.3.2, Eq. S15: Shouldn't there be a $\int dx x$ in the numerator of the right-hand side? The left side is an expectation value of x, so I think there should be an average over it on the right side.

Author response. This is correct. Fixed.

Reviewer comment 2.19

17. Supplement Sec. 5.1, fifth line: Doesn't the model assumption $\langle r \rangle = nR/N$ contradict the point the authors just made in Sec. 4.3.2, that the read frequency $\langle r \rangle/N$ is not an unbiased estimator of the actual frequency n/N?

Author response. Thank you for point out this issue, which requires further clarification. This issue is related to the Reviewer comment 2.10 and has to do with the imprecision of the language that we used in the main text. If the measurement process is unbiased, then, for any given lineage, its frequency estimated from read counts in expectation equals its actual frequency in the population, i.e. $\frac{\langle r \rangle}{R} = \frac{n}{N}$. This is in fact true (see Figure 4A). But if we consider all lineages whose barcode frequency is r/R, their expected frequency in the population (conditional on the read count r) is not r/R. We have revised the language in the main text to clarify this issue (LL 232–251).

Reviewer comment 2.20

18. Figure S10: Why not plot this data as scatter points of individual mutants, with inferred fitness from one replicate vs. inferred fitness from the other replicate?

Author response. We cannot do that because different barcoded subpopulations acquire adaptive mutations in different biological replicates.

Reviewer comment 2.21

19. Supplement references: There is some inconsistent formatting here (capitalization in titles, e.g., "dna," and abbreviation/capitalization of journal titles), which the journal probably won't fix themselves.

Author response. We went through our bibliography and fixed all the issues that we found.

Reviewer comment 2.22

20. Finally, I appreciated the food-themed acronyms and wondered if the authors would consider the title "Putting BASIL on a BLT."

Author response. This is very clever! We decided to follow the reviewer's suggestion and changed the title.
